# Crystallization-induced mechanofluorescence for visualization of polymer crystallization

**DOI:** 10.1038/s41467-020-20366-y

**Published:** 2021-01-05

**Authors:** Sota Kato, Shigeki Furukawa, Daisuke Aoki, Raita Goseki, Kazusato Oikawa, Kousuke Tsuchiya, Naohiko Shimada, Atsushi Maruyama, Keiji Numata, Hideyuki Otsuka

**Affiliations:** 1 Department of Chemical Science and Engineering, Tokyo Institute of Technology, 2-12-1 Ookayama, Meguro-ku, Tokyo 152-8550 Japan; 2 Biomacromolecules Research Team, RIKEN Center for Sustainable Resource Science, 2-1 Hirosawa, Wako, Saitama 351-0198 Japan; 3 School of Life Science and Technology, Tokyo Institute of Technology, 4259 Nagatsuta-cho, Midori-ku, Yokohama, Kanagawa 226-8501 Japan

**Keywords:** Polymers, Polymer characterization, Polymers

## Abstract

The growth of lamellar crystals has been studied in particular for spherulites in polymeric materials. Even though such spherulitic structures and their growth are of crucial importance for the mechanical and optical properties of the resulting polymeric materials, several issues regarding the residual stress remain unresolved in the wider context of crystal growth. To gain further insight into micro-mechanical forces during the crystallization process of lamellar crystals in polymeric materials, herein, we introduce tetraarylsuccinonitrile (TASN), which generates relatively stable radicals with yellow fluorescence upon homolytic cleavage at the central C–C bond in response to mechanical stress, into crystalline polymers. The obtained crystalline polymers with TASN at the center of the polymer chain allow not only to visualize the stress arising from micro-mechanical forces during polymer crystallization via fluorescence microscopy but also to evaluate the micro-mechanical forces upon growing polymer lamellar crystals by electron paramagnetic resonance, which is able to detect the radicals generated during polymer crystallization.

## Abstract (web summary)

Residual stress in crystalline polymers can lead to material failure, but currently methods which can quantify residual stress in such materials are lacking. Here, by using fluorescent-type mechanochromophores, the authors develop a method to visualize and quantify low degrees of stress that arises from micro-mechanical forces during polymer crystallization.

## Introduction

Crystalline polymers such as polyethylene and polyethylene terephthalate are indispensable for our daily lives. Their mechanical and optical properties strongly depend on their crystallinity^1^, which in turn is closely correlated to molding parameters. The crystallization of such synthetic polymers has been extensively studied, especially from a morphological and kinetic perspective^2^. Unlike amorphous resins, crystalline polymers experience stress at freezing and contain residual stress due to crystallization. However, the micro-mechanical forces during polymer crystallization remains an intensely debated topic, since suitable techniques to quantitatively evaluate this phenomenon have not yet been established. So far, only theoretical, indirect-experimental, or empirical discussions have been employed. In order to avoid material failure due to micro-mechanical forces during polymer crystallization, researchers and manufacturers have employed various experimental techniques. The approaches to assess the magnitude of micro-mechanical forces during polymer crystallization in polymer components can be classified into three distinct categories: Non-destructive^3^, destructive^4^, and predictive^5^. Among these, non-destructive methods such as holographic interferometry and synchrotron X-ray diffraction analysis have been used to visualize the physical relaxation of components during heating and to calculate their initial stress state. Despite the fact that the non-destructive nature of these methods is without doubt an advantage, they have been unable to accomplish neither a qualitative evaluation nor stress visualization in real time. From the viewpoint of the molecular topology of the amorphous phase, micro-mechanical forces during polymer crystallization in tie chain segments, which are molecular connections between individual crystallites, have been yet an important issue for the tensile or compression behavior of semicrystalline polymers, the fracture toughness, and the resistance to slow crack growth^6,7^. Topological features such as tie chain segments and loop segments, and the alternation of crystalline and amorphous domains have formed the basis of most molecular-level description of the semicrystalline state^8,9^.

**Fig. 1:**
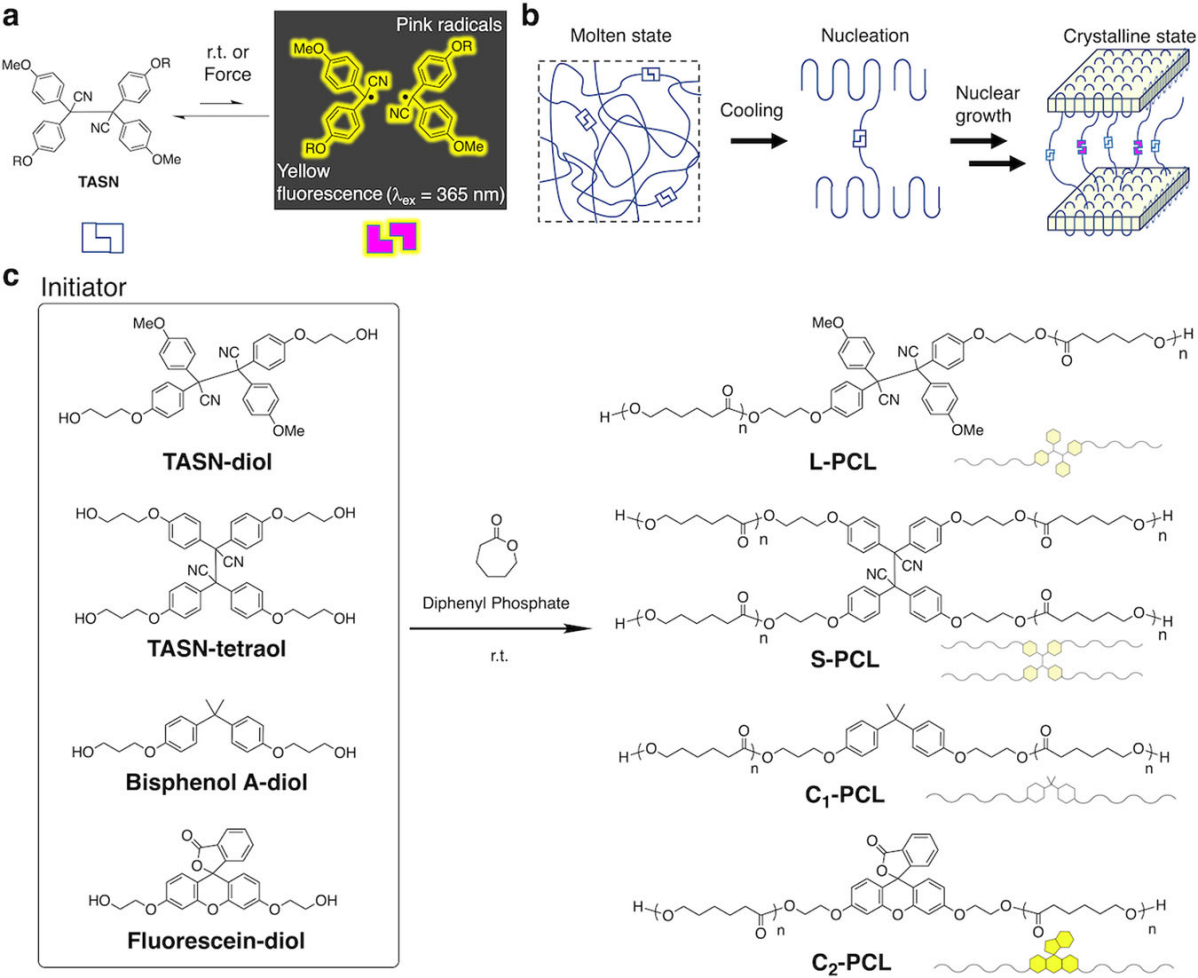
Conceptual illustration of the TASN moiety as a fluorescent probe and schematic image of CIMF. **a** Chemical structure of tetraarylsuccinonitrile (TASN) and its equilibrium with the corresponding C–C-cleaved radicals. **b** Schematic image of the crystallization of a TASN-centered polymer from its molten state. **c** Synthetic scheme of TASN-containing linear polycaprolactone (**L-PCL**), TASN-containing star polycaprolactone (**S-PCL**), and control polycaprolactones that contain bisphenol A (**C**_**1**_**-PCL**) or fluorescein (**C**_**2**_**-PCL**) at the center of the chain.

On the other hand, various types of mechano-functional polymers have recently been developed based on mechano-responsive molecules called mechanophores^10–12^, which are activated in response to mechanical stress arising from exposure to ultrasonic^13–16^, stretching^17–19^, compression^20^, swelling^21^, or solvent-coagulation forces^22–25^. Our group has been developing radical-type mechanochromophores that exhibit coloration and fluorescence derived from relatively stable radicals generated upon homolytic cleavage of C–C bonds in response to exposure to mechanical stress. For example, a diarylbibenzofuranone^22^ generates blue radicals upon the homolytic cleavage of its central C–C bond, while tetraarylsuccinonitrile (TASN; Fig. 1a)^26^ generates pink radicals that emit yellow fluorescence under UV irradiation.

In the present study, the above-mentioned radical-type mechanochromophores, especially TASN, were introduced into the center of the main chain of crystalline polymers. As TASN at the cross-linking point can be cleaved in response to the solvent-coagulation force in freezing cross-linked polymer gels, we hypothesized that TASN in a tie molecule could potentially be mechanically cleaved by the growth of lamellar crystals (henceforth: crystallization-induced mechanofluorescence (CIMF)) (Fig. 1b). Accordingly, the corresponding fluorescence of radicals generated from TASN would enable a visualization of the crystallization. The advantage of using radical-type mechanochromophores lies in the generated radicals, which can be examined by electron paramagnetic resonance (EPR) measurements. Thus, the present system is suitable for the detection of low degrees of stress that arise from micro-mechanical forces during polymer crystallization. Fluorescence microscopy observations of CIMF allows the precise identification of the stress location and an in-depth clarification of the polymer crystallization process.

## Results

### Strategy and system

In this study, polycaprolactone (PCL) was chosen as the crystalline polymer based on the following three considerations. Firstly, even at room temperature, TASN is in equilibrium with a small amount of dissociated radicals, and it is unstable at temperatures beyond 100 °C. The melting point (~50 °C) and the crystallization temperature (~30 °C) of PCL mean that TASN can be used under relatively mild conditions, which the effect of this paper is limited to PCL melts. Secondly, a series of PCLs can be easily synthesized in one step using the living ring-opening polymerization of ε-caprolactone initiated by TASN derivatives with two (**TASN-diol**) or four hydroxy groups **(TASN-tetraol)** as the initiators. Thirdly, PCL has been studied extensively and ample basic data on PCL is available^27–35^.

As shown in Fig. 1c, a series of PCLs were synthesized using (1) **TASN-diol**, (2) **TASN-tetraol**, (3) **Bisphenol A-diol**, and (4) **Fluorescein-diol** as initiators, in the presence of diphenyl phosphate (DPP) as the bulk catalyst^36,37^. Linear and star-shaped PCLs with TASN are henceforth referred to as **L-PCL** and **S-PCL**, respectively. Control PCLs prepared from **Bisphenol A-diol** and **Fluorescein-diol** are henceforth referred to as **C**_**1**_**-PCL** and **C**_**2**_**-PCL**, respectively. **C**_**1**_**-PCL** was synthesized as a control PCL that is not cleaved in response to micro-mechanical forces during polymer crystallization, while **C**_**2**_**-PCL** was synthesized as a polymer that continuously emits fluorescence during the formation of spherulites. All PCLs were successfully synthesized and their molecular weight, molecular weight distribution, and thermal properties are summarized in Table 1. PCLs with different molecular weights were successfully synthesized by changing the monomer-to-initiator feed ratio.

**Table 1:** Molecular weight and thermal properties of L-PCL, S-PCL, C_1_-PCL, and C_2_-PCL Molecular weight and thermal properties of the synthesized linear and star-shaped polycaprolactones that contain a mechanochromophore or a control moiety.

Structure	[I]:[M]	*M*_n_^a^/g mol^−1^	*M*_n_^b^/g mol^−1^	*M*_w_/*M*_n_^b^	*T*_m_^c^/°C	Δ*H*_m_^c^/J g^−1^	*X*_c_^c^/%
L-PCL	1:50	7100	9800	1.16	51	59	44
	1:100	13,000	17,500	1.19	55	57	42
	1:200	24,400	24,100	1.20	55	57	42
S-PCL	1:100	12,600	14,400	1.13	51	54	40
	1:200	25,200	29,500	1.14	55	58	43
C_1_-PCL	1:100	11,800	14,900	1.07	55	60	44
C_2_-PCL	1:100	8600	10,500	1.19	51	61	45

Conditions for the diphenyl phosphate (DPP)-catalyzed polymerizations.

Initiator: ε-caprolactone [I]; monomer: [M]; [DPP]/[I]=2; ambient temperature.

^a^Determined by ^1^H NMR spectroscopy.

^b^Determined by GPC (eluent: THF; calibration: polystyrene standards).

^c^DSC data refer to the second heating cycle; heating rate: 10 °C min^−1^;

*T*_m_: melting temperature; Δ*H*_m_: melting enthalpy; *X*_c_: crystallinity (*X*_c_ = Δ*H*_m_/Δ*H*°_m_).

Δ*H*°_m_: melting enthalpy of perfect PCL crystals (135.44 J g^–1^)^27^.

### CIMF during isothermal crystallization

**Fig. 2:**
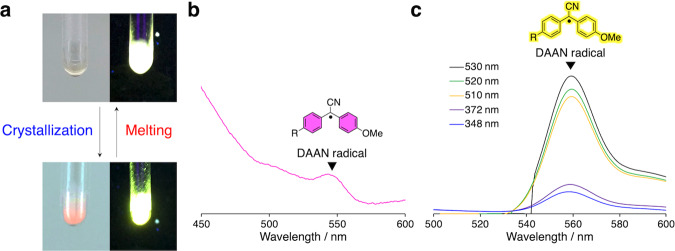
CIMF during isothermal crystallization. **a** CIMF of **L-PCL** (*M*_n,NMR_ = 13000), which contains a TASN moiety at the center of the main chain. **b** Solid-state UV-vis spectrum of **L-PCL** (*M*_n,NMR_ = 13,000) after isothermal crystallization. **c** Solid-state fluorescence spectra of **L-PCL** (*M*_n,NMR_ = 13,000) after isothermal crystallization under different excitation wavelength. (DAAN radical: diarylacetonitrile radical derived from TASN).

CIMF was investigated on the series of synthesized PCLs. Initially, the PCL samples were exposed to simple heating. During the heating process (heating rate: 50 °C min^−1^), the PCL samples were completely molten at 70 °C, where they were kept for 2 min. Subsequently, the samples were cooled to 30 °C (cooling rate: –20 °C min^−1^), where they were kept for crystallization. During the crystallization, both pink coloration and yellow fluorescence, due to the dissociation of TASN, were observed (Fig. 2a). During the cooling process from 70 °C to 0 °C, EPR measurements on **L-PCL** (*M*_n,NMR_ = 13,000) supported the hypothesis that the dissociation of TASN is mainly induced by crystallization and not by melting (Supplementary Fig. 21). Solid-state UV-vis (Fig. 2b) and fluorescence measurements (Fig. 2c) on **L-PCL** (*M*_n,NMR_ = 13,000) after isothermal crystallization showed an absorption peak at 548 nm in the UV-vis measurement and an emission peak at 560 nm in the fluorescence measurement. These peaks unambiguously indicate the generation of diarylacetonitrile (DAAN) radicals derived from TASN^26^. Furthermore, in the solid-state fluorescence measurement, the fluorescence intensity drastically increased upon shifting the excitation wavelength to 548 nm. Therefore, an argon laser (*λ*_ex_ = 514 nm) was chosen for the subsequent fluorescent microscopy observations of PCLs in order to gain clear images of the spherulites.

### Quantitative evaluation of the amount of radicals generated during CIMF

**Fig. 3:**
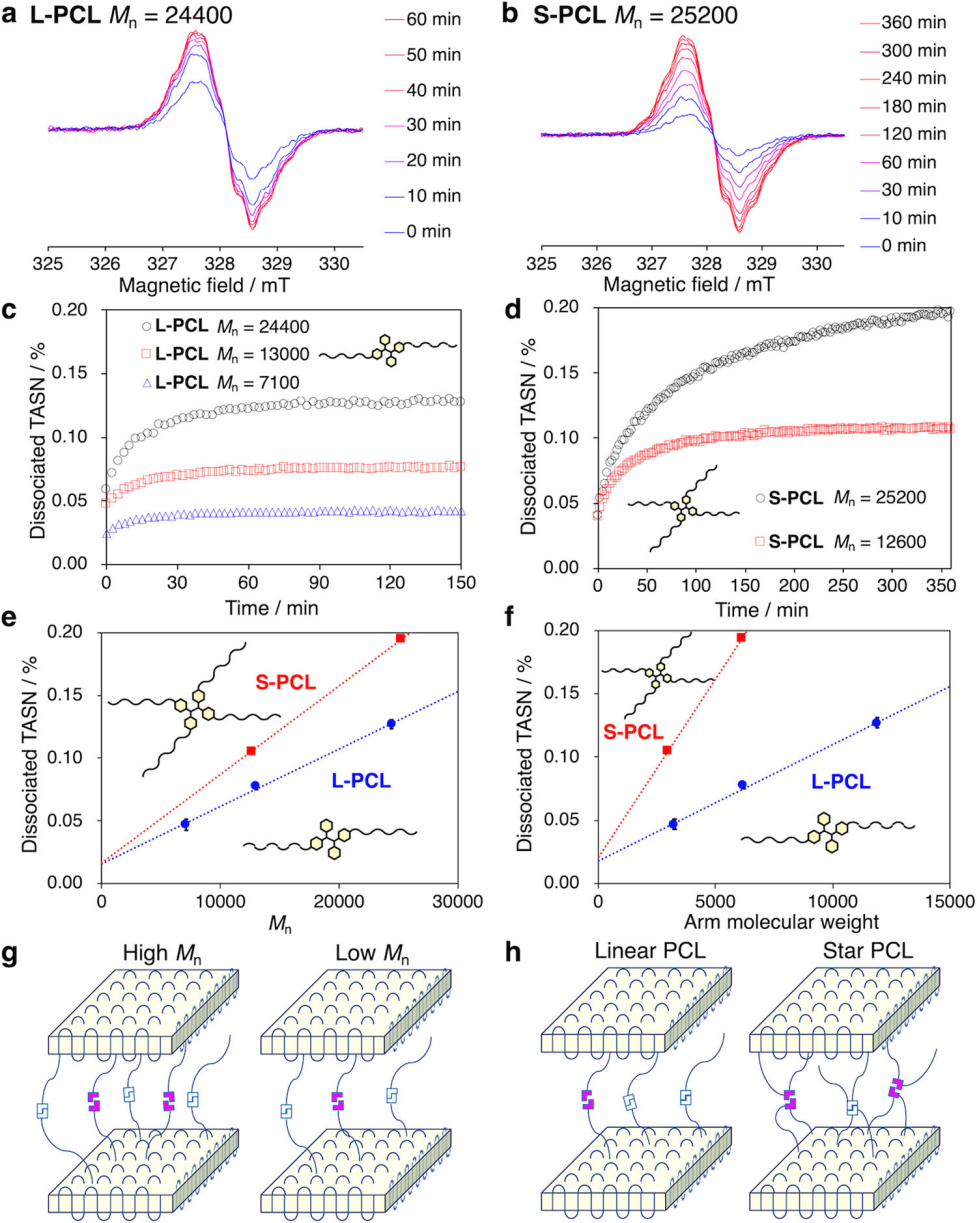
Generation of radicals during isothermal crystallization. EPR spectra of (**a**) **L-PCL** (*M*_n,NMR_ = 24400) and (**b**) **S-PCL** (*M*_n,NMR_ = 25,200) during isothermal crystallization. Dissociated TASN (%) in (**c**) **L-PCL** (*M*_n,NMR_ = 7100: black circle, 13,000: red square, and 24,400: blue triangle) and (**d**) **S-PCL** (*M*_n,NMR_ = 12,600: black circle, and 25,200: red square) during isothermal crystallization. **e** Relationship between molecular weight and dissociated TASN (%) of **L-PCL** (*M*_n,NMR_ = 7100, 13,000, and 24,400; blue circle and line) and **S-PCL** (*M*_n,NMR_ = 12,600 and 25,200; red square and line) during isothermal crystallization. **f** Linear relationship between the molecular weight of the arm and dissociated TASN (%) of **L-PCL** (*M*_n,NMR_ = 7100, 13,000, and 24,400; blue circle and line) and **S-PCL** (*M*_n,NMR_ = 12,600 and 25,200; red square and line) during isothermal crystallization. Plausible schematic illustration of chain conformations of (**g**) high- and low-molecular-weight polymers and (**h**) linear and star-shaped crystalline polymers between lamellar structures.

The generation of radicals during the isothermal crystallization was examined for the **L-PCL**s and **S-PCL**s. In the present system, the mechanical force induced by crystallization can be evaluated by EPR measurements of the generated radicals. We speculate that the knowledge of unpaired spin density may allow quantitative estimates of segmental forces by assuming that the dissociated radicals from TASN moieties can be detected without oxidation, which is controlled by the sealing of the capillary after being degassed. The concentration of the radicals formed from the cleavage of TASN was determined by comparing the area of the observed integral spectrum with a 0.01-mM solution of 4-hydroxy-2,2,6,6-tetramethylpiperidin-1-oxyl (TEMPOL) in benzene under the same experimental conditions. The PCL samples were fully molten at 70 °C, and subsequently cooled to 30 °C (cooling rate: –20 °C min^−1^) for crystallization. Figure 3a, b show the EPR spectra of **L-PCL** (*M*_n_ = 24,400) and **S-PCL** (*M*_n_ = 25,200), respectively, during the crystallization at 30 °C. The calculated *g* value (2.003) in both cases indicates that the generated radicals are carbon-centered. The signal intensity generated from TASN increases with time. Fig. 3c, d show the dissociation ratios of TASN calculated from the EPR spectra in Fig. 3a, b, respectively. The TASN dissociation ratio reached an equilibrium after 90 minutes for **L-PCL** and after 330 min for **S-PCL**. It should be noted that the time to reach the equilibrium approximately doubled when the molecular weight doubled^38–41^.

Figure 3e, f show the plots of the dissociation ratios of the TASN moiety for the PCL samples as a function of the molecular weight and molecular weight of the arm, respectively. These data suggest (1) an increase in the dissociation ratio of the TASN moiety with increasing molecular weight and (2) a large difference in the dissociation ratio of the TASN moiety due to the difference in primary structures (linear or star-shaped polymers). A plausible mechanism to rationalize the experimentally observed results could be based on the following considerations: (1) with increasing molecular weight in the same crystallinity (Table 1), the fraction of TASN moieties between crystallites^6,7,42–44^, and hence the fraction of ‘active’ TASN moieties, i.e., moieties dissociated in response to mechanical stress induced by crystallization would increase (Fig. 3g). (2) When comparing the topology between linear and star-shaped polymers that contain arms of identical molecular weight (Fig. 3f), **S-PCL** polymers have more arms involved in the formation of lamellar layers, which would lead to a further increase of ‘active’ TASN moieties that serve as tie molecules compared to **L-PCL** polymers (Fig. 3h). The mechano-activation behavior strongly depends on the environment of the mechanophore^11^. In solution under ultrasonication, single mechanophores at the center of the main chain are more likely to be activated in polymers with high molecular length^45,46^. On the other hand, in the bulk state under exposure to grinding, the mechano-responsiveness of mechanochromophores, especially radical-type mechanophores such as TASN, is governed by both the molecular weight and the number of branches^47,48^. The insights into mechano-responsiveness in polymer crystallization obtained in this paper are expected to broaden the understanding of polymer mechanochemistry.

### Fluorescence microscopy measurements of CIMF

The visualization of the crystallization process, which is the most important part of this paper, was performed by confocal laser scanning microscopy (CLSM). So far, the twisting behavior of lamellar crystals in spherulites^49–52^ has been examined by polarized light microscopy^38,53^, small-angle light scattering^54^, transmission electron microscopy^30^, scanning electron microscopy^55^, and atomic force microscopy^29,56,57^. However, reports on the residual stress of the twisting behavior during the growth of spherulites remain elusive. Here, we addressed this dearth by converting the micro-mechanical forces during polymer crystallization into fluorescence arising from dissociated TASN radicals generated during the growth of the spherulites.

**Fig. 4:**
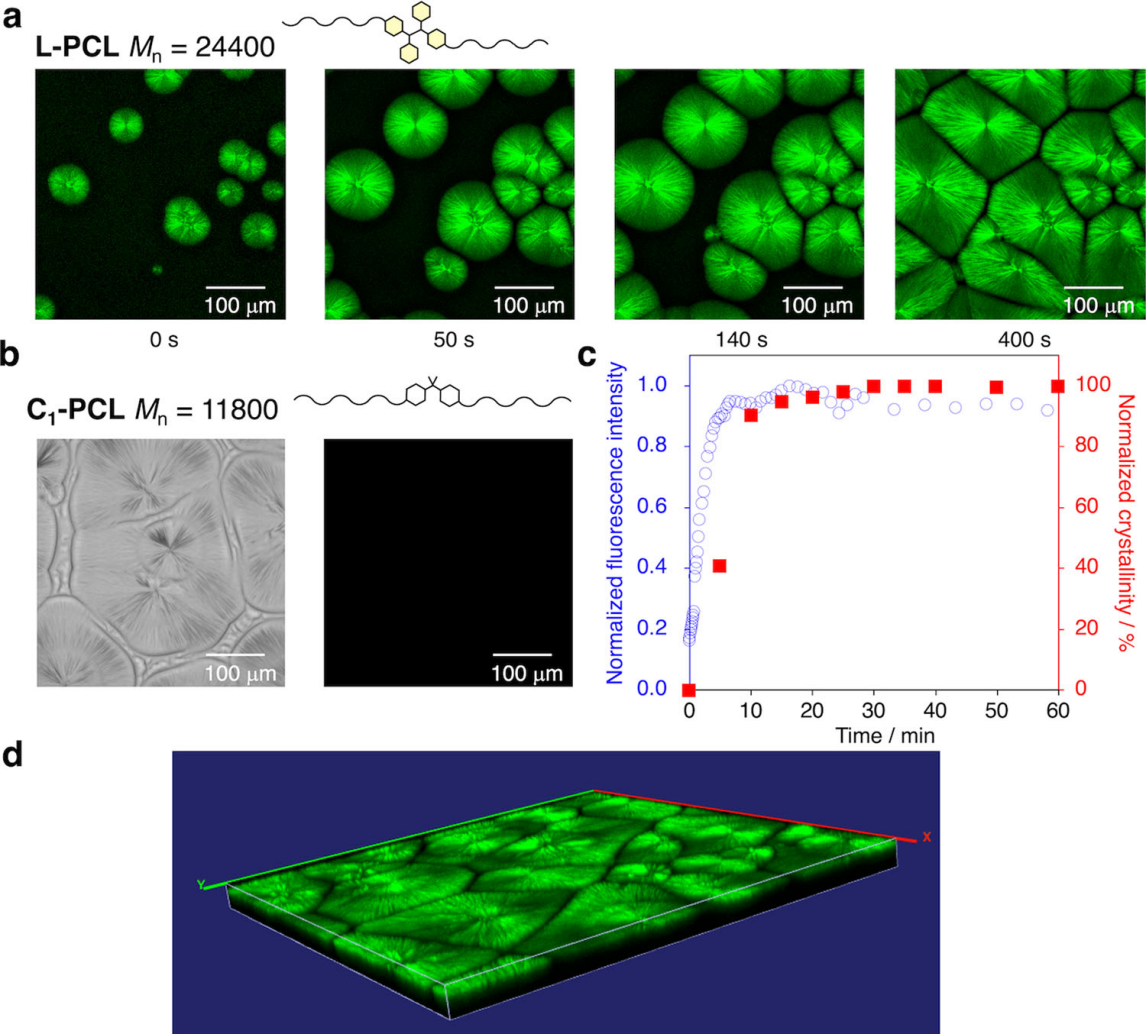
Visualization of micro-mechanical forces during isothermal crystallization. **a** Microscopic images of **L-PCL** (*M*_n_ = 24,400) captured by confocal laser scanning microscopy (CLSM; *λ*_ex_ = 514 nm; scale bar: 100 μm) during isothermal crystallization. **b** Microscopic images of **C-PCL** (*M*_n_ = 11,800) under bright-field conditions (left) and under excitation (right; *λ*_ex_ = 514 nm; scale bar: 100 μm) during isothermal crystallization. **c** Normalized fluorescence intensity (blue open circles) and normalized crystallinity (red closed squares) during isothermal crystallization for **L-PCL** (*M*_n_ = 24,400). **d** 3D image of **L-PCL** (*M*_n_ = 24,400) alpha mode captured by CLSM (*λ*_ex_ = 514 nm; scale bar: 100 μm) after isothermal crystallization.

As shown in Fig. 4a (Supplementary Movie 1), microscopy images of **L-PCL** (*M*_n_ = 24,400) recorded at 30 °C reveal that spherulites grow from the molten state. Furthermore, a comparison with the bright-field images (Supplementary Fig. 22) demonstrated that the fluorescence emission is observed only in the growth direction of the lamellar crystals, while **C**_**1**_**-PCL**, which does not contain TASN moieties, exhibited no fluorescence (Fig. 4b). In contrast, **C**_**2**_**-PCL**, which contains a fluorescein moiety, exhibited fluorescence in both the crystalline and the amorphous regions (Supplementary Fig. 27). As shown in Fig. 4c, the fluorescence intensity increased during isothermal crystallization with increasing crystallinity, which was estimated based on DSC measurements under identical thermal conditions. The strong relationship between the fluorescence intensity and the crystallinity indicates that the stress generated during the growth of the spherulites can be evaluated at the molecular level. This tool could thus be used to evaluate micro-mechanical forces during polymer crystallization in the field of crystal growth, and also as a guideline for the molecular design of advanced materials.

We also confirmed that the distribution of the skewness and kurtosis in the fluorescence region (Supplementary Fig. 23) decreased with the growth of the lamellar crystals, which indicates that the spherulites are growing uniformly in two dimensions. CLSM also enables scanning the spherulites in 3D scan mode^58^ (Fig. 4d, Supplementary Figs. 25 and 26) as well as in airyscan mode (Supplementary Fig. 24), which allows detailed observations on the growth of lamellar crystals. It should be noted that the direct visualization of polymer crystallization with mechano-activated fluorescent radicals offers unprecedented insight into crystal growth processes.

## Discussion

Radical-type mechanofluorophores such as tetraarylsuccinonitrile (TASN) allow us to evaluate the micro-mechanical forces during polymer crystallization in crystalline polymers by electron paramagnetic resonance (EPR) spectroscopy and to visualize it by fluorescence microscopy. The radical-generating behavior and the real-time stress visualization have revealed the relationship between crystalline polymer architecture and the efficiency of the force transmission during the growth of spherulites. The increase of molecular weight of the main chain affects the dissociation of TASN at tie molecules, whereby star-shaped polycaprolactone contain more micro-mechanical forces during polymer crystallization at tie molecules than linear polycaprolactones. This strategy can be expected to be applicable to a wide variety of polymers in order to evaluate their micro-mechanical forces during polymer crystallization, which may ultimately lead to design guidelines for advanced materials. We are convinced that this report on crystallization-induced mechanofluorescence (CIMF) will open research avenues in polymer and materials sciences.

## Methods

### Preparation of polymers containing TASN moiety

The synthesis, purification, and characterization, which includes the NMR, IR, DSC and GPC data, mechanical characterization, control experiments, and additional details for all polymers, are described in detail in Supplementary Information.

### Evaluations of dissociated TASN radicals during polymer crystallization

Variable-temperature EPR measurements were carried out on a JEOL JES-X320 X-band EPR spectrometer equipped with a JEOL DVT temperature controller. The samples were filled to 5 mm glass capillaries, and the capillaries were sealed after being degassed. In case of isothermal crystallization, the glass capillaries were heated to 70 °C to melt the crystalline polymer absolutely, then the sample was kept at 30 °C (cooling rate: –20 °C min^−1^) and measured. The spectra of samples were measured using microwave power of 0.1 mW and a field modulation of 0.1 mT with a time constant of 0.03 s and a sweep rate of 0.25 mTs^−1^. The concentration of the radicals formed from the cleavage of TASN was determined by comparing the area of the observed integral spectrum with a 0.01 mM solution of 4-hydroxy-2,2,6,6-tetramethylpiperidin-1-oxyl (TEMPOL) in benzene under the same experimental conditions. The Mn^2+^ signal was used as an auxiliary standard.

### CLSM experiments to observe crystallization-induced mechanofluorescence

The expression of mechano-activated fluorescence from TASN moiety at the center of the main chain was visualized by a Zeiss LSM880 with Airyscan (Carl Zeiss, Oberkochen, Germany) with ×20 objective lens using 514 nm excitation and 526–651 nm emission wavelengths for **L-PCL**, **S-PCL**, and **C**_**1**_**-PCL**. For isothermal crystallization, the samples placed on the cover glass (18 mm × 18 mm) were heated to 70 °C, then the sample was kept at 30 °C. As a control sample, **C**_**2**_**-PCL** was visualized using 488 nm excitation and 506–647 emission wavelengths. All samples were prepared by spin coater MSC-200D (Japan Create Co., Ltd.). The spin coating conditions are as following; polymer concentration: 120 mg/mL, rotational speed of disk: 800 rpm, 30 s, solvent: 1,2-dichloroethane.

## Supplementary information

Supplementary Information

Description of Additional Supplementary Files

Supplementary Movie 1

## Data Availability

All data supporting the findings of this study are available within the article and its Supplementary Information. All other data are available from the corresponding author upon reasonable request.
