# Supplementary material for: Crystallization-induced mechanofluorescence for visualization of polymer crystallization
Source: Nat Commun. 2021 Jan 5;12:126. doi: 10.1038/s41467-020-20366-y (PMC7785725; doi:10.1038/s41467-020-20366-y)
Supplement: Supplementary file 1 — Supplementary Information [file 41467_2020_20366_MOESM1_ESM.pdf]

Supplementary Information

Crystallization-induced Mechanofluorescence for Visualization of Polymer Crystallization

Sota Kato, [1] Shigeki Furukawa, [1] Daisuke Aoki, [1] Raita Goseki, [1] Kazusato Oikawa, [2] Kousuke Tsuchiya, [2] Naohiko Shimada, [3] Atsushi Maruyama, [3] Keiji Numata, [2] and Hideyuki Otsuka [1*]

E-mail: otsuka@polymer.titech.ac.jp

[1]Department of Chemical Science and Engineering, Tokyo Institute of Technology, 2-12-1 Ookayama, Meguro-ku, Tokyo 152-8550, Japan

[2]Biomacromolecules Research Team, RIKEN Center for Sustainable Resource Science, 2-1 Hirosawa, Wako, Saitama 351-0198, Japan

[3]School of Life Science and Technology, Tokyo Institute of Technology, 4259 Nagatsuta-cho, Midori-ku, Yokohama, Kanagawa 226-8501, Japan

Materials and Methods

Materials:

All solvents and reagents were purchased from Sigma-Aldrich, Wako Pure Chemical Industries, Tokyo Chemical Industry, and Kanto Chemical, and used as received, unless otherwise noted. 1,4-Dioxane was distilled under reduced pressure over calcium hydride.

Instruments:

^{1}H and ^{13}C NMR spectra were recorded on a Bruker AVANCE Ⅲ HD500 spectrometer. IR spectra were recorded on a JEOL FT/IR-4100 Fourier transform infrared spectrometer as thin films with KBr and cast on NaCl. Analytical gel permeation chromatographic (GPC) measurements were carried out at 40 °C on TOSOH HLC-8320 GPC system equipped with a guard column (TOSOH TSK guard column Super H-L), three columns (TOSOH TSK gel SuperH 6000, 4000, and 2500), a differential refractive index detector, and a UV-vis detector. Tetrahydrofuran (THF) was used as the eluent at a flow rate of 0.6 mL/min. Polystyrene (PS) standards (M_n = 4430-3242000; M_w/M_n = 1.03−1.08) were used to calibrate the GPC system.

Measurements:

Variable-temperature electron paramagnetic resonance (EPR) spectroscopy

Variable-temperature EPR measurements were carried out on a JEOL JES-X320 X-band EPR spectrometer equipped with a JEOL DVT temperature controller.

The samples were filled to 5 mm glass capillaries, and the capillaries were sealed after being degassed. The glass capillaries were heated at 70 °C to melt the crystalline polymer absolutely, and the sample was cooled from 70 °C to 0 °C to measure every 10 °C. In case of isothermal crystallization, the glass capillaries were heated to 70 °C, then the sample was kept at 30 °C and measured. The spectra of samples were measured using microwave power of 0.1 mW and a field modulation of 0.1 mT with a time constant of 0.03 s and a sweep rate of 0.25 mTs^{-1}.

The concentration of the radicals formed from the cleavage of TASN was determined by comparing the area of the observed integral spectrum with a 0.01 mM solution of 4-hydroxy-2,2,6,6-tetramethylpiperidin-1-oxyl (TEMPOL) in benzene under the same experimental conditions. The Mn^{2+} signal was used as an auxiliary standard.

The g value was calculated according to the following equation:

$$g = h\nu / \beta H$$

where h is the Planck constant, ν is the microwave frequency, β is the Bohr magneton and H is the magnetic field. The equilibrium of TASN was assumed to simply involve the associated TASN and the dissociated radicals, without irreversible side reactions; under this assumption, the ratio of dissociated TASN was calculated from the peak intensity, by using TEMPOL as a standard.

CLSM experiments to observe crystallization-induced mechanofluorescence

The expression of mechano-activated fluorescence from TASN moiety at the center of the main chain was visualized by a Zeiss LSM880 with Airyscan (Carl Zeiss, Oberkochen, Germany) with 20x objective lens using 514 nm excitation and 526-651 nm emission wavelengths for **L-PCL**, **S-PCL**, and **C_1-PCL**. For isothermal crystallization, the samples placed on the cover glass (18 mm × 18 mm) were heated to 70 °C, then the sample was kept at 30 °C. As a control sample, **C_2-PCL** was visualized using 488 nm excitation and 506-647 emission wavelengths. All samples were prepared by spin coater MSC-200D (Japan Create Co., Ltd.). The spin coating conditions are as following; polymer concentration: 120 mg / mL, rotational speed of disk: 800 rpm, 30 seconds, solvent: 1,2-dichloroethane.

Synthesis of TASN-diol and BPA-diol:

Compound **1**, **TASN-diol** and **BPA-diol** were synthesized according to previously published methods.[1,2]

Synthesis of 2,2-bis(4-methoxyphenyl)acetonitrile (**2**):

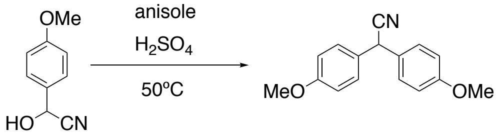

1 **2**

In a 200 mL two necked round bottom flask, 35% sulfuric acid (14.7 mL), compound **1** (9.79 g, 60.0 mmol), and anisole (15.7 mL) were added, and the mixture was allowed to stir at 50 °C for 24 h. After cooling to room temperature, the remaining liquid was decanted and the residual solid was dissolved in ethyl acetate. The organic layer was washed with brine and water, dried over Na_2SO_4, and evaporated. The residue was recrystallized from a mixed solvent of THF and ethanol, filtrated, and dried in vacuo to give compound **2** as a white powder (11.4 g, 75%). ^{1}H NMR (500 MHz, $CDCl_3$): δ/ppm 7.23 (d, J = 8.7 Hz, 4H, aromatic), 6.87 (d, J = 8.7 Hz, 4H, aromatic), 5.05 (s, 1H, -*CH*(CN)-), 3.79 (s, 6H, –O*CH₃*); ^{13}C NMR (125 MHz, $CDCl_3$): δ/ppm 159.38, 128.82, 128.30, 120.20, 114.50, 55.36, 41.05. FT-IR (KBr, cm^{-1}): 3004, 2965, 2935, 2897, 2244, 1610, 1583, 1510, 1458, 1304, 1255, 1176, 1121, 1028, 972, 845, 833, 808, 772, 600

Synthesis of 2,2-bis(4-hydroxyphenyl)acetonitrile (**3**):

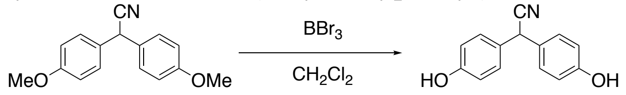

2 **3**

In a round bottom flask, a solution of compound **2** (5.07 g, 20.0 mmol) in dry methylene chloride (112 mL) was formed. With stirring, boron tribromide (5.69 mL, 60.0 mmol) was added dropwise in ice bath over 15 min. Then, the mixture was allowed to stir for 5 h as it warmed to room temperature. The reaction mixture was poured into ice water and the precipitate collected by filtration was dried in vacuo to give compound **3** as a white powder (4.45 g, 99%) ^{1}H NMR (500 MHz, DMSO-d_6): δ/ppm 9.67 (s, 2H, -Ph*OH*), 7.14 (d, J = 8.4 Hz, 4H, aromatic), 6.76 (d, J = 8.4 Hz, 4H, aromatic), 5.47 (s, 1H, -*CH*(CN)-); ^{13}C NMR (125 MHz, DMSO-d_6): δ/ppm 157.42, 129.00, 127.82, 121.57, 116.17, 40.17. FT-IR (KBr, cm^{-1}): 3268, 2263, 1597, 1509, 1456, 1375, 1245, 826, 596, 545.

Synthesis of 2,2-bis(4-(3-hydroxypropoxy)phenyl)acetonitrile (**4**):

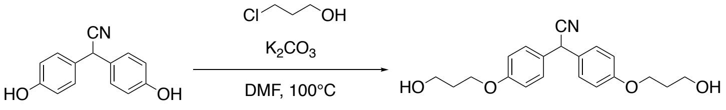

3 **4**

In a round bottom flask, a mixture of compound **3** (4.47 g, 18.7 mmol), potassium carbonate (15.5 g, 112 mmol), and DMF (94.0 mL) was heated to 100 °C for 30 min. 3-Chloro-1-propanol (9.30 mL, 112 mmol) was added to the reaction mixture and the reaction held at 100 °C for 2 h. Once cooled to room temperature and filtered, the mixture was extracted with ethyl acetate, washed with water and brine, and then dried over Na_2SO_4. After filtration and evaporation, the crude product was purified by column chromatography on silica gel eluting with chloroform and dried

in vacuo to give compound **4** as white powder (3.41 g, 50% yield). ^{1}H NMR (500 MHz, $CDCl_3$): δ/ppm 7.21 (d, *J* = 8.4 Hz, 4H, aromatic), 6.88 (d, *J* = 8.4 Hz, 4H, aromatic), 5.03 (s, 1H, -$\mathit{C\underline{H}}$(CN)-), 4.09 (t, *J* = 6.2 Hz, 4H, $–CH_2\mathit{C\underline{H}_2}OH$), 3.83 (q, *J* = 5.5 Hz, 4H, – $\mathit{C\underline{H}_2}CH_2CH_2OH$), 2.13 (t, 2H, $–CH_2\mathit{O\underline{H}}$), 2.02 (quin, 4H, – $CH_2\mathit{C\underline{H}_2}CH_2OH$); ^{13}C NMR (125 MHz, $CDCl_3$): δ/ppm 158.62, 128.83, 128.36, 120.17, 115.03, 65.56, 60.03, 41.00, 31.90. FT-IR (KBr, cm^{-1}): 3268, 2263, 1597, 1509, 1456, 1375, 1245, 826, 596, 545. FAB-MS (m/z): [M+]+ calcd for $C_{20}H_{23}NO_4$, 341.1627; found, 341.1635.

Synthesis of TASN-tetraol:

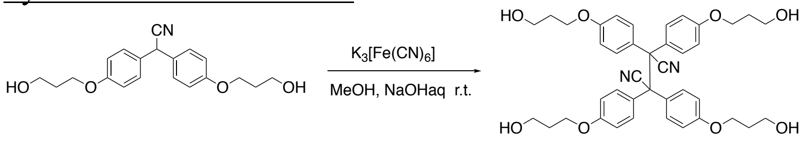

4 TASN-tetraol

In a round bottom flask, a mixture of compound **4** (2.60 g, 7.62 mmol) and methanol (61.0 mL) was formed. With stirring, potassium ferricyanide (2.76 g, 8.38 mmol) in 5 M NaOH aqueous solution (12.2 mL) was added dropwise in water bath. Then, the mixture was allowed to stir for 10 minutes at room temperature. After filtration, the product was washed with water. After the crude solid was dissolved in chloroform and was reprecipitated in hexane for three times, the resulting precipitate was filtered and dried in vacuo to give **TASN-tetraol** as a white powder (2.13 g, 82% yield). ^{1}H NMR (500 MHz, $CDCl_3$): δ/ppm 7.05 (d, *J* = 8.8 Hz, 8H, aromatic), 6.92 (d, *J* = 8.8 Hz, 8H, aromatic), 4.59 (t, 4H, $–CH_2\mathit{O\underline{H}}$), 4.03 (t, *J* = 6.2 Hz, 8H, $–CH_2\mathit{C\underline{H}_2}OH$), 3.55 (q, *J* = 5.5 Hz, 8H, – $\mathit{C\underline{H}_2}CH_2CH_2OH$), 1.85 (quin, 8H, – $CH_2\mathit{C\underline{H}_2}CH_2OH$); ^{13}C NMR (125 MHz, DMSO-d_6): δ/ppm 158.94, 131.15, 129.05, 121.67, 114.38, 65.06, 58.21, 57.63, 32.47. FT-IR (KBr, cm^{-1}): 3367, 2952, 2881, 1607, 1579, 1560, 1541, 1509, 1473, 1418, 1396, 1297, 1256, 1188, 1125, 1058, 1013, 991, 949, 827, 797, 628, 541. ESI-MS (m/z): [M+Na]+ calcd for $C_{40}H_{44}N_2O_8$, 703.2989; found, 703.2992.

Synthesis of Fluorescein-diol:

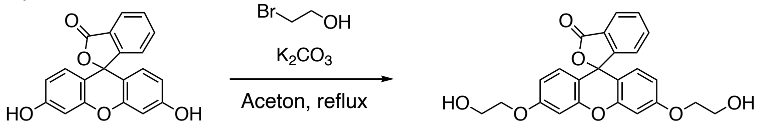

In a round bottom flask, a mixture of fluorescein (0.251 g, 0.75 mmol), potassium carbonate (0.260 g, 1.88 mmol), and acetone (20 mL) was stirred at r.t. for 1 h. 2-Bromoethanol (0.215 mL, 3.0 mmol) was added to the reaction mixture, and the reaction was held at 60 °C in reflux for 24 h. Once cooled to r.t. and filtered, the filtrate was extracted with dichrolomethane, washed with water and brine, and then dried over Na_2SO_4. After filtration and evaporation, the crude product was purified by column chromatography on silica gel eluting with ethyl chroloform/methanol (95/5, v/v) and dried in vacuo to give **Fluorescein-diol** as orange solid (0.21 g, 68% yield).

^{1}H NMR (500 MHz, $CDCl_3$): δ/ppm 8.28 (d, *J* = 8.2 Hz, 1H, aromatic), 7.76 (t, *J* = 7.7 Hz, 1H, aromatic), 7.69 (t, *J* = 7.7 Hz, 1H, aromatic), 7.31 (d, *J* = 7.5 Hz, 1H, aromatic), 6.98 (d, *J* = 2.4 Hz, 1H, aromatic), 6.92 (d, *J* = 8.9 Hz, 1H, aromatic), 6.87 (d, *J* = 9.7 Hz, 1H, aromatic), 6.78 (d, *J* = 8.9 Hz, 1H, aromatic), 6.55 (d, *J* = 9.6 Hz, 1H, aromatic), 6.46 (d, *J* = 1.9 Hz, 1H, aromatic), 4.20 (t, *J* = 4.6 Hz, 2H, $–CH_2\mathit{C\underline{H}_2}OH$), 4.12 (m, 2H, $–CH_2\mathit{C\underline{H}_2}OH$), 4.03 (br, 2H, –

$OC\underline{H_2}CH_2$–), 3.55 (br, 2H, –$OC\underline{H_2}CH_2$–), 2.00 (br, 1H, –$CH_2\underline{OH}$), 1.20 (br, 1H, –$CH_2\underline{OH}$); ^{13}C NMR (125 MHz, $CDCl_3$): δ/ppm 165.29, 164.91, 160.24, 155.03, 155.00, 134.05, 132.72, 131.23, 130.98, 130.30, 129.87, 129.44, 127.86, 116.80, 114.94, 114.55, 104.01, 100.67, 78.07, 70.45, 66.40, 59.87, 59.24, 31.34, 29.30, 22.29, 13.04. FT-IR (NaCl, cm^{-1}): 3353, 2925, 2855, 2360, 1716, 1668, 1644, 1594, 1506, 1471, 1384, 1260, 1041, 919, 854, 802, 755, 706, 666, 614. ESI-MS (m/z): [M+Na]+ calcd for $C_{24}H_{20}O_7$, 443.1101; found, 443.1095.

Synthesis of TASN-containing linear polycaprolactone (L-PCL):

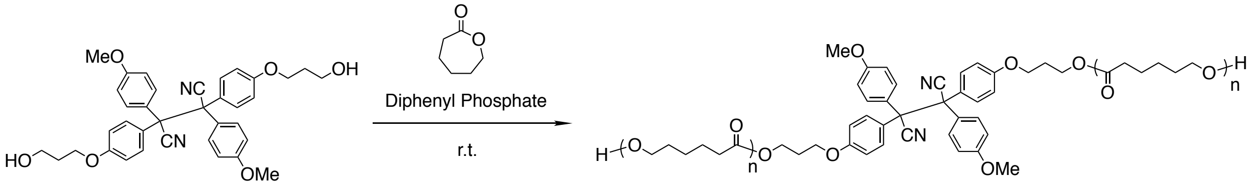

TASN-diol derivative (59.3 mg, 0.100 mmol) and diphenyl phosphate (50.0 mg, 0.200 mmol) were added to a 10 mL schlenk flask and freeze drying was carried out. ε-Caprolactone (1.10 mL, 10.0 mmol) was added to the flask, after that the solution was kept with stirring at room temperature for 24 h. The reaction mixture was diluted with chloroform and the solution was precipitated in methanol, filtered, and dried under vacuum to afford a white powder (1.10 g, 92% yield). The M_n value was determined by ^1H NMR spectrum and M_w/M_n value was determined by analytical GPC with polystyrene standards. M_n = 13000 g/mol, M_w/M_n = 1.19.

Linear PCLs of different molecular weight were synthesized in similar manners as mentioned above. TASN-diol : ε-caprolactone = 1 : 50 and 1 : 200 [mol], M_n = 7100 and 24400 g/mol, M_w/M_n = 1.16 and 1.20. ^1H NMR (500 MHz, $CDCl_3$) : δ/ppm 7.18-7.15 (m, 8H, aromatic), 6.77-6.75 (m, 8H, aromatic), 4.06 (br, CH_2), 3.79 (s, 6H, OCH_3), 2.31 (br, CH_2), 2.11 (quin, J = 6 Hz, 4H, CH_2), 1.65 (br, CH_2), 1.37 (br, CH_2)

Synthesis of TASN-containing star polycaprolactone (S-PCL):

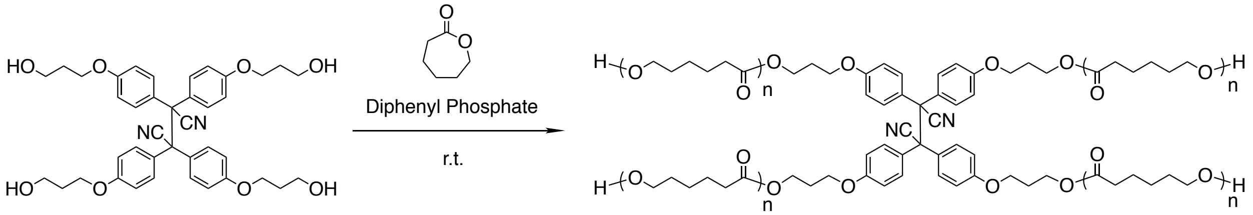

TASN-tetraol derivative (68.1 mg, 0.100 mmol) and diphenyl phosphate (50.0 mg, 0.200 mmol) were added to a 10 mL schlenk flask and freeze drying was carried out. ε-Caprolactone (1.10 mL, 10.0 mmol) was added to the flask, after that the solution was kept with stirring at room temperature for 24 h. The reaction mixture was diluted with chloroform and the solution was precipitated in methanol, filtered, and dried under vacuum to afford a white powder (1.05 g, 88% yield). The M_n value was determined by ^1H NMR spectrum and M_w/M_n value was determined by analytical GPC with polystyrene standards. M_n = 12600 g/mol, M_w/M_n = 1.13.

Star PCLs of different molecular weight were synthesized in similar manners as mentioned above. TASN-tetraol : ε-caprolactone = 1 : 200 [mol], M_n = 25200 g/mol, M_w/M_n = 1.14. ^1H NMR (500 MHz, $CDCl_3$) : δ/ppm 7.16-7.14 (m, 8H, aromatic), 6.75-6.73 (m, 8H, aromatic), 4.06 (br, CH_2), 3.79 (s, 6H, OCH_3), 2.31 (br, CH_2), 2.11 (quin, J = 6 Hz, 8H, CH_2), 1.64 (br, CH_2), 1.37 (br, CH_2)

Synthesis of Bisphenol A-containing linear polycaprolactone (C_1-PCL):

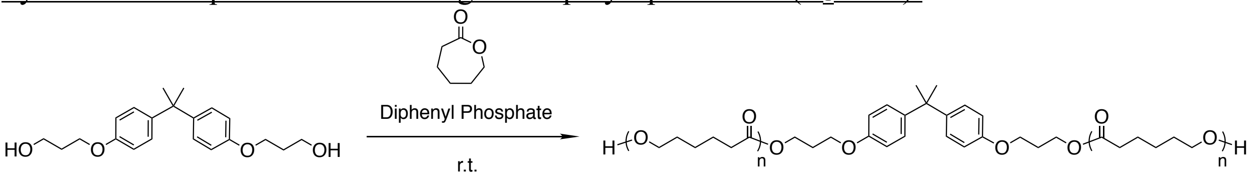

BPA-diol derivative (103 mg, 0.300 mmol) and diphenyl phosphate (150 mg, 0.600 mmol) were added to a 10 mL schlenk flask and freeze drying was carried out. ε-Caprolactone (3.32 mL, 30.0 mmol) was added to the flask, after that the solution was kept with stirring at room temperature for 24 h. The reaction mixture was diluted with chloroform and the solution was precipitated in methanol, filtered, and dried under vacuum to afford a white powder (3.08 g, 87% yield). The M_n value was determined by ^{1}H NMR spectrum and M_w/M_n value was determined by analytical GPC with polystyrene standards. M_n = 11800 g/mol, M_w/M_n = 1.07. ^{1}H NMR (500 MHz, $CDCl_3$) : δ/ppm 7.13-7.11 (m, 4H, aromatic), 6.79-6.76 (m, 4H, aromatic), 4.06 (br, CH_2), 2.31 (br, CH_2), 2.09 (quin, J = 6 Hz, 4H, CH_2), 1.64 (br, CH_2), 1.38 (br, CH_2)

Synthesis of Fluorescein-containing linear polycaprolactone (C_2-PCL):

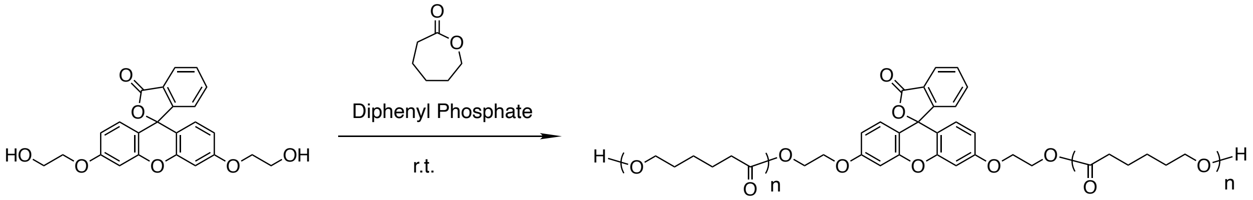

Fluorescein-diol derivative (21.1 mg, 0.05 mmol) and diphenyl phosphate (25.0 mg, 0.100 mmol) were added to a 10 mL schlenk flask and freeze drying was carried out. ε-Caprolactone (0.550 mL, 5.00 mmol) was added to the flask, after that the solution was kept with stirring at room temperature for 24 h. The reaction mixture was diluted with chloroform and the solution was precipitated in methanol, filtered, and dried under vacuum to afford a yellow powder (0.65 g, 78% yield). The M_n value was determined by ^{1}H NMR spectrum and M_w/M_n value was determined by analytical GPC with polystyrene standards. M_n = 8600 g/mol, M_w/M_n = 1.19. ^{1}H NMR (500 MHz, $CDCl_3$) : δ/ppm 7.18 (d, J = 8.0 Hz, 4H, aromatic), 7.11 (t, J = 7.9 Hz, 2H, aromatic), 4.06 (br, CH_2), 2.31 (br, CH_2), 1.64 (br, CH_2), 1.38 (br, CH_2)

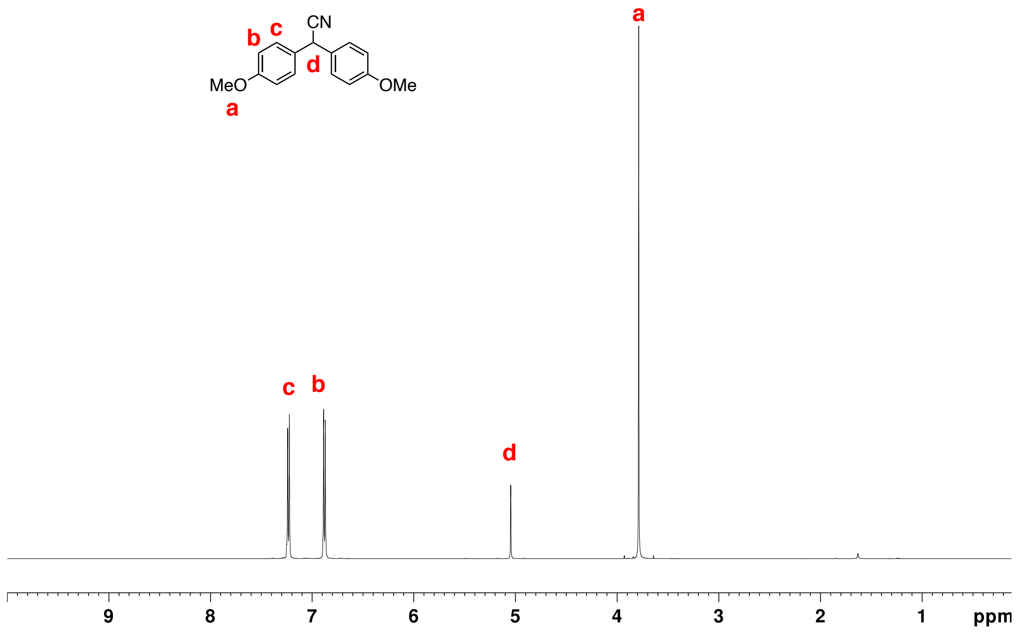

Supplementary Fig. 1.

^{1}H NMR spectrum of **2**. ($CDCl_3$, 500 MHz)

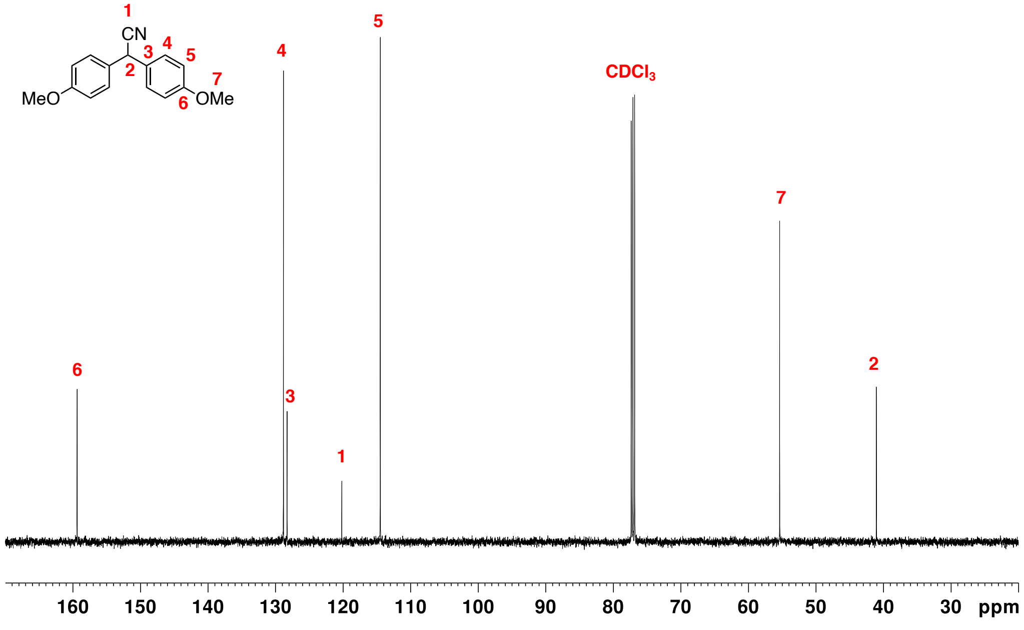

Supplementary Fig. 2.

^{13}C NMR spectrum of **2**. ($CDCl_3$, 125 MHz)

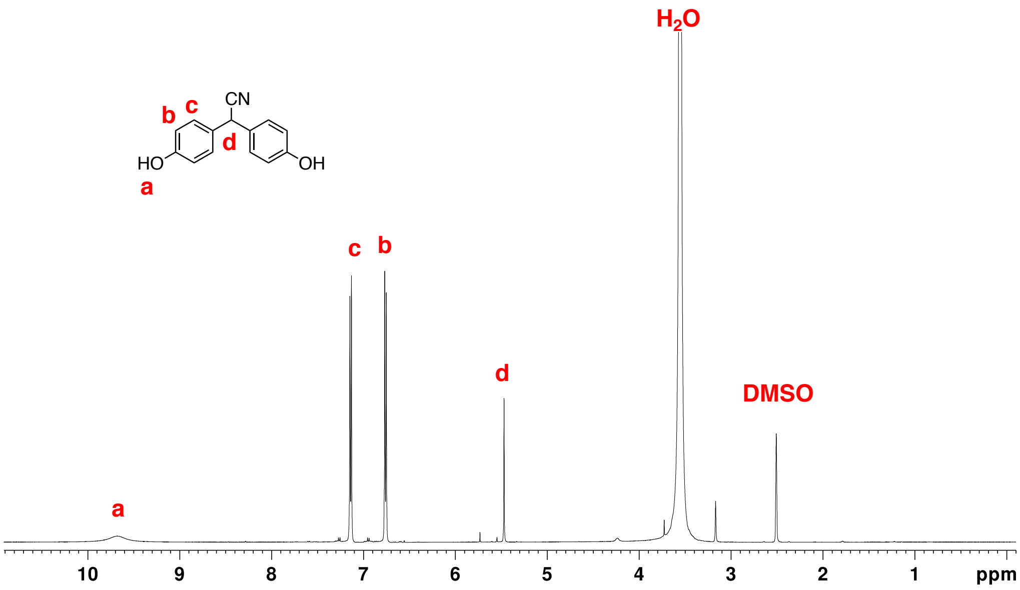

Supplementary Fig. 3.
^{1}H NMR spectrum of **3**. (DMSO-d_6, 500 MHz)

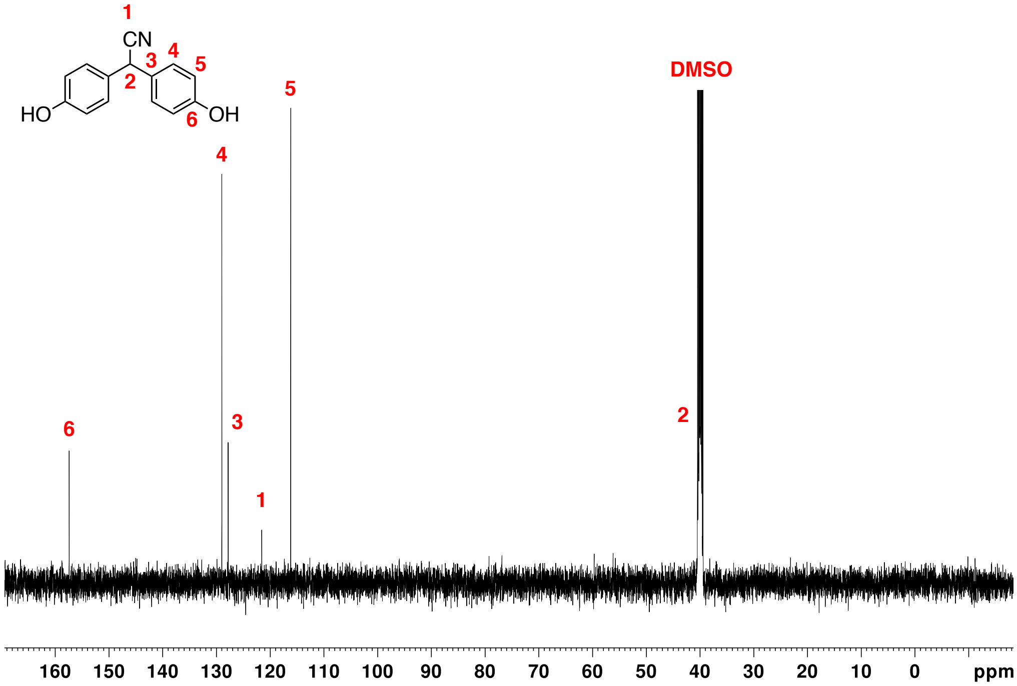

Supplementary Fig. 4.
^{13}C NMR spectrum of **3**. (DMSO-d_6, 125 MHz)

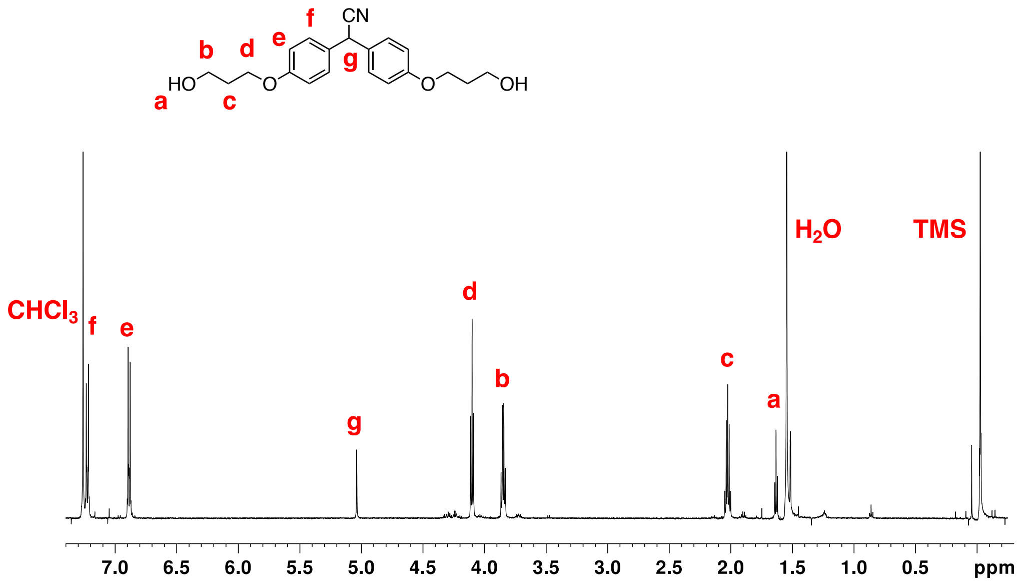

Supplementary Fig. 5.
^{1}H NMR spectrum of **4**. ($CDCl_3$, 500 MHz)

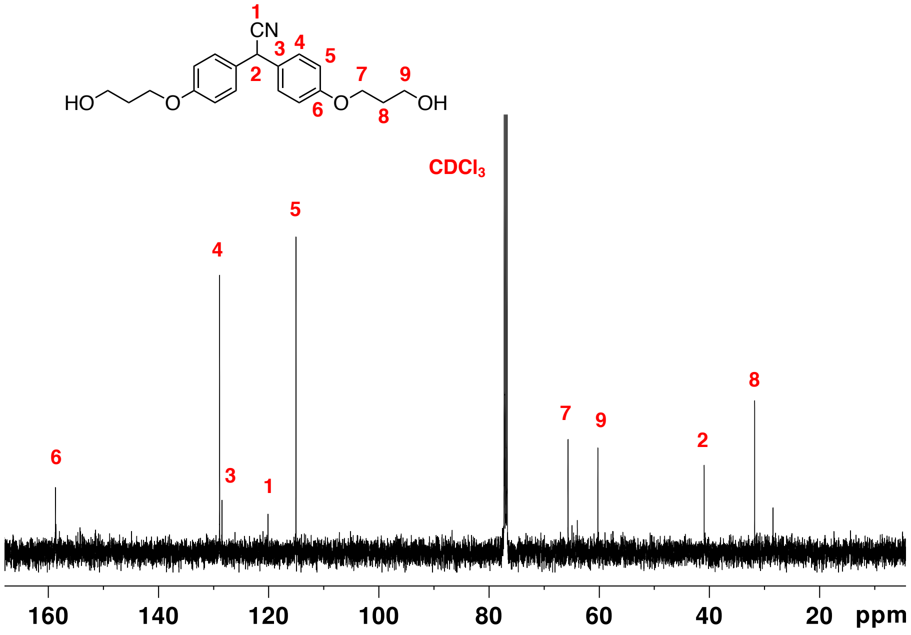

Supplementary Fig. 6.
^{13}C NMR spectrum of **4**. ($CDCl_3$, 125 MHz)

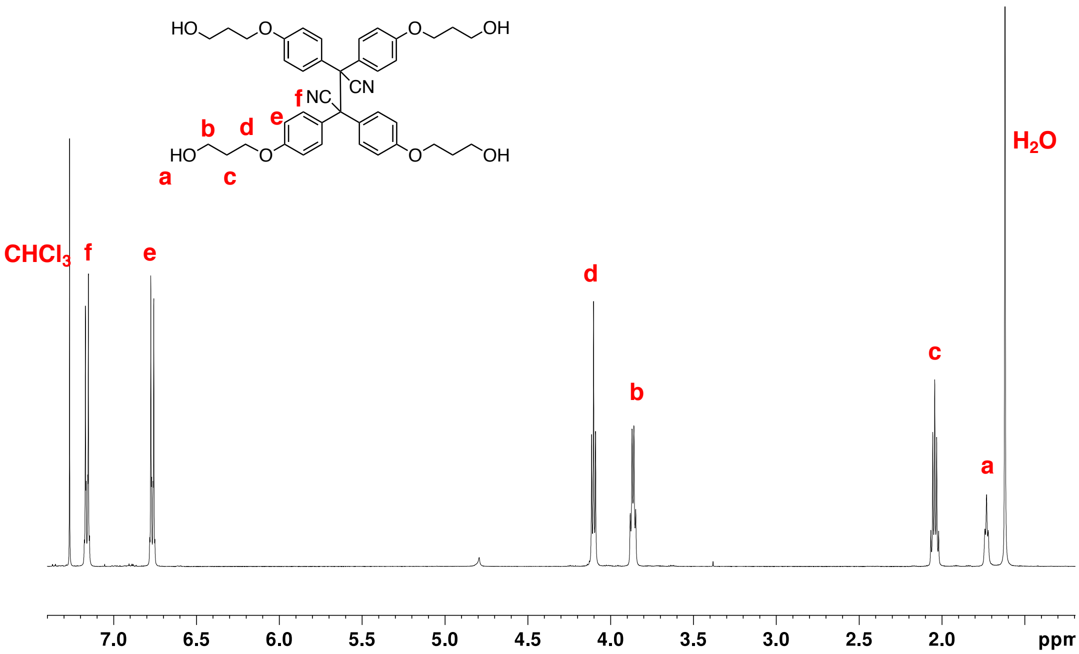

Supplementary Fig. 7.
^{1}H NMR spectrum of **TASN-tetraol**. ($CDCl_3$, 500 MHz)

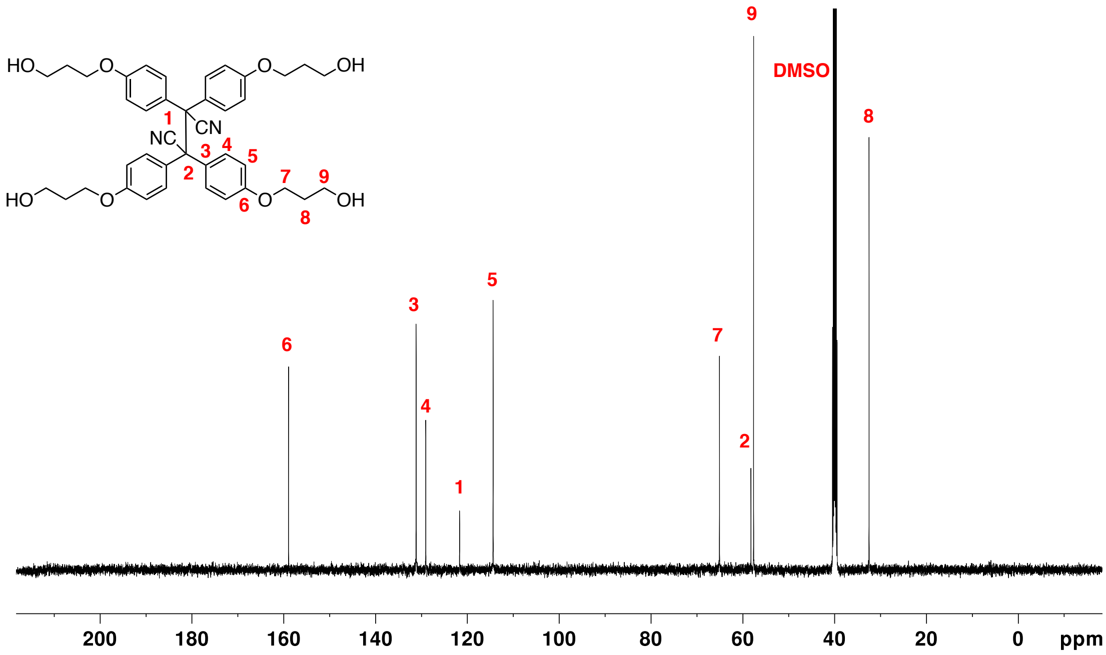

Supplementary Fig. 8.
^{13}C NMR spectrum of **TASN-tetraol**. (DMSO-d_6, 125 MHz)

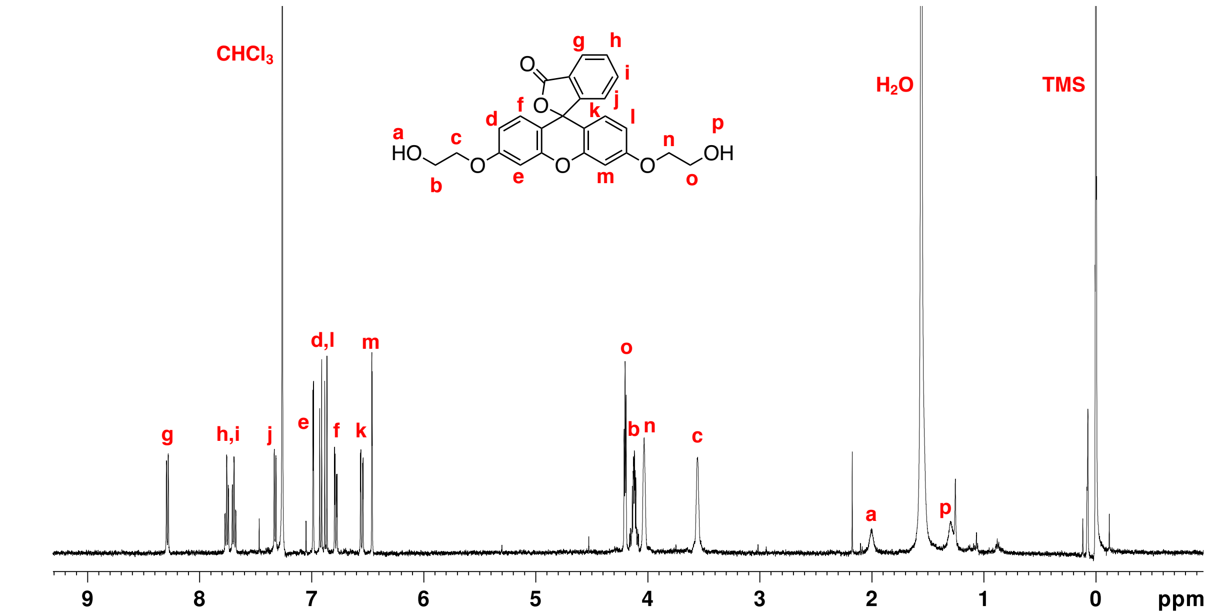

Supplementary Fig. 9.

^{1}H NMR spectrum of **Fluorescein-diol**. ($CDCl_3$, 500 MHz)

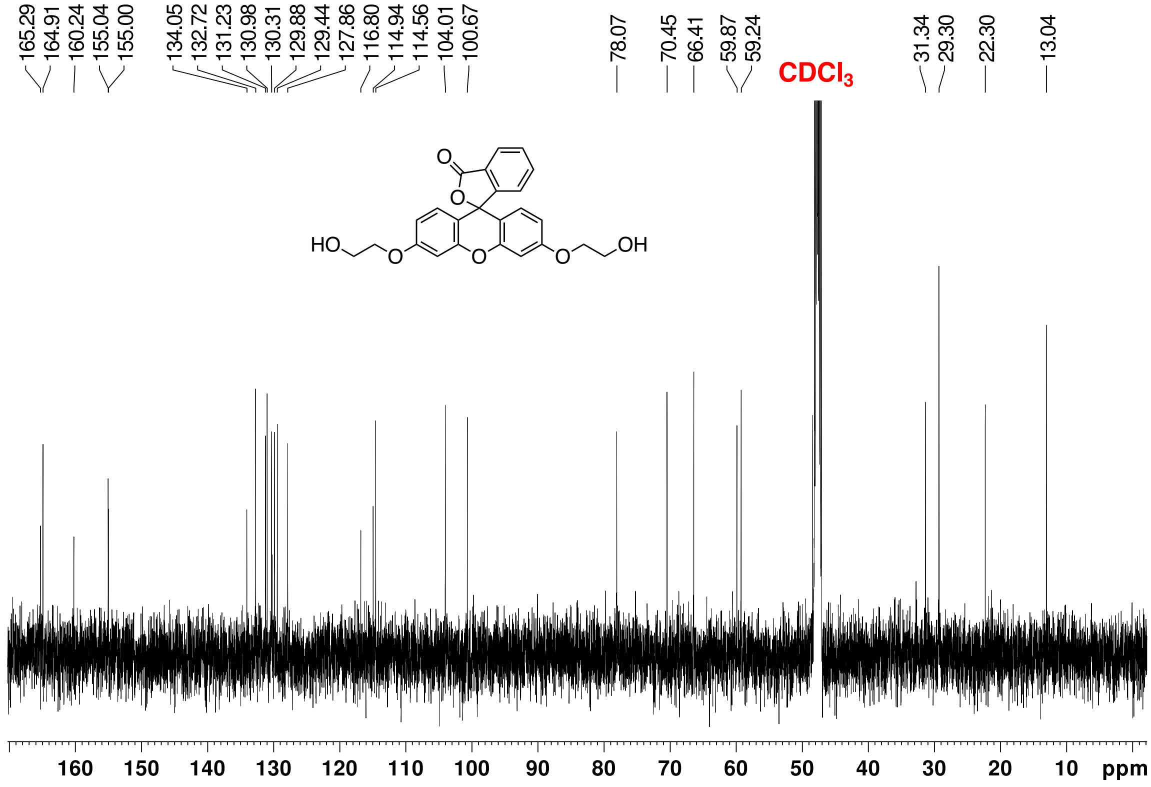

Supplementary Fig. 10.

^{13}C NMR spectrum of **Fluorescein-diol**. ($CDCl_3$, 125 MHz)

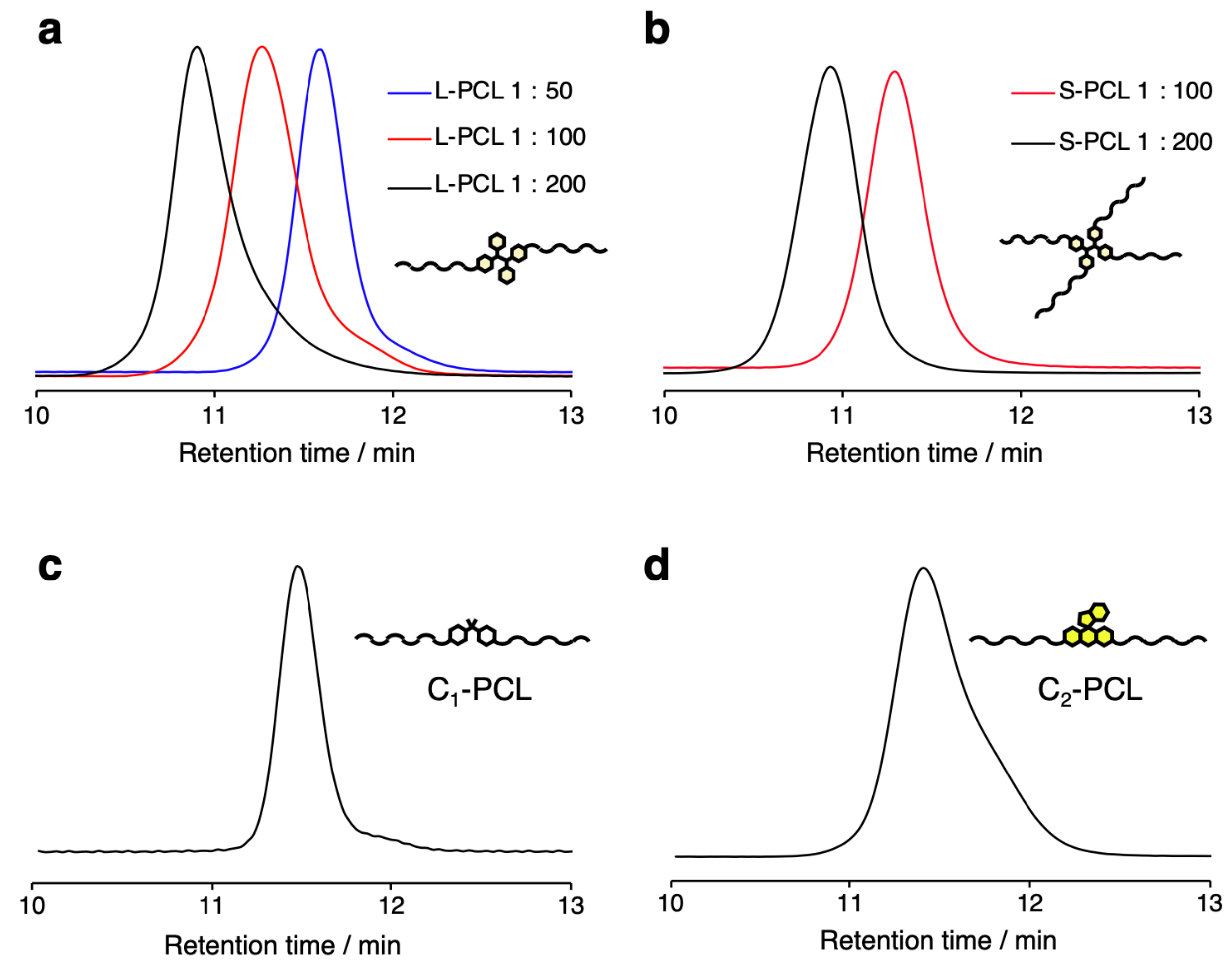

Supplementary Fig. 11.

GPC curves of crystalline polymer. GPC curves of (**a**) **L-PCL**, (**b**) **S-PCL**, (**c**) **C_1-PCL** and (**d**) **C_2-PCL** (PS standard, THF).

Supplementary Table 1.

Feed ratio and molecular weight of L-PCL, S-PCL, C_1-PCL, and C_2-PCL. Molecular weight of the synthesized linear and star-shaped polycaprolactones that contain a mechanochromophore or a control moiety calculated by ^{1}H NMR spectra (Figure S2-S8).

Structure	$[I]_{feed}$: $[M]_{feed}$	e / f	$[I]_{NMR}$: $[M]_{NMR}$	$M_{n,NMR}$ / g mol^{-1}
L-PCL	1 : 50	14.3	1 : 57.3	7100
	1 : 100	27.2	1 : 108.6	13000
	1 : 200	52.3	1 : 209.0	24400
S-PCL	1 : 100	26.2	1 : 104.7	12600
	1 : 200	53.6	1 : 214.5	25200
C_1-PCL	1 : 100	50.5	1 : 101.0	11800
C_2-PCL	1 : 100	140.3	1 : 70.2	8600

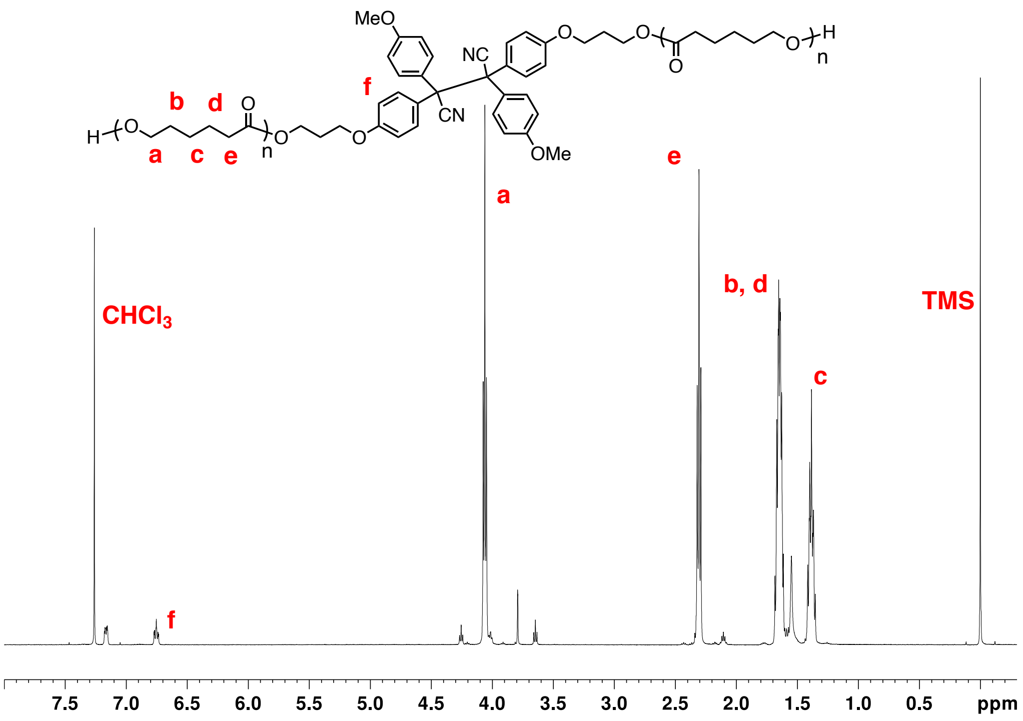

Supplementary Fig. 12.
^{1}H NMR spectrum of L-PCL ($M_{n,NMR}$ = 7100, M_w/M_n = 1.16). ($CDCl_3$, 500 MHz)

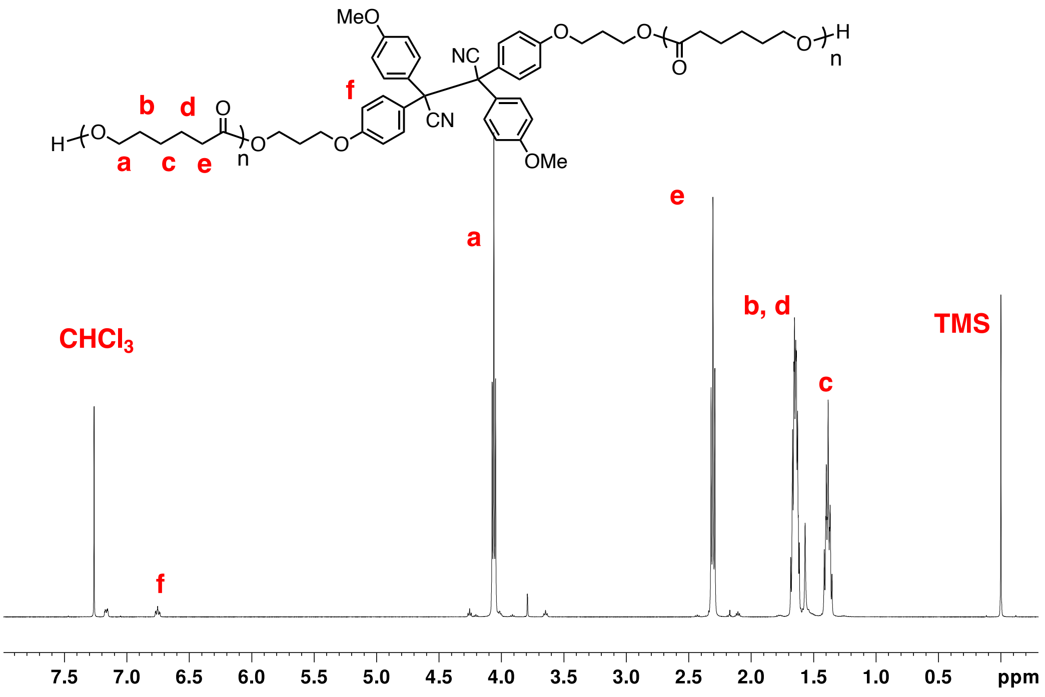

Supplementary Fig. 13.
^{1}H NMR spectrum of L-PCL ($M_{n,NMR}$ = 13000, M_w/M_n = 1.19). ($CDCl_3$, 500 MHz)

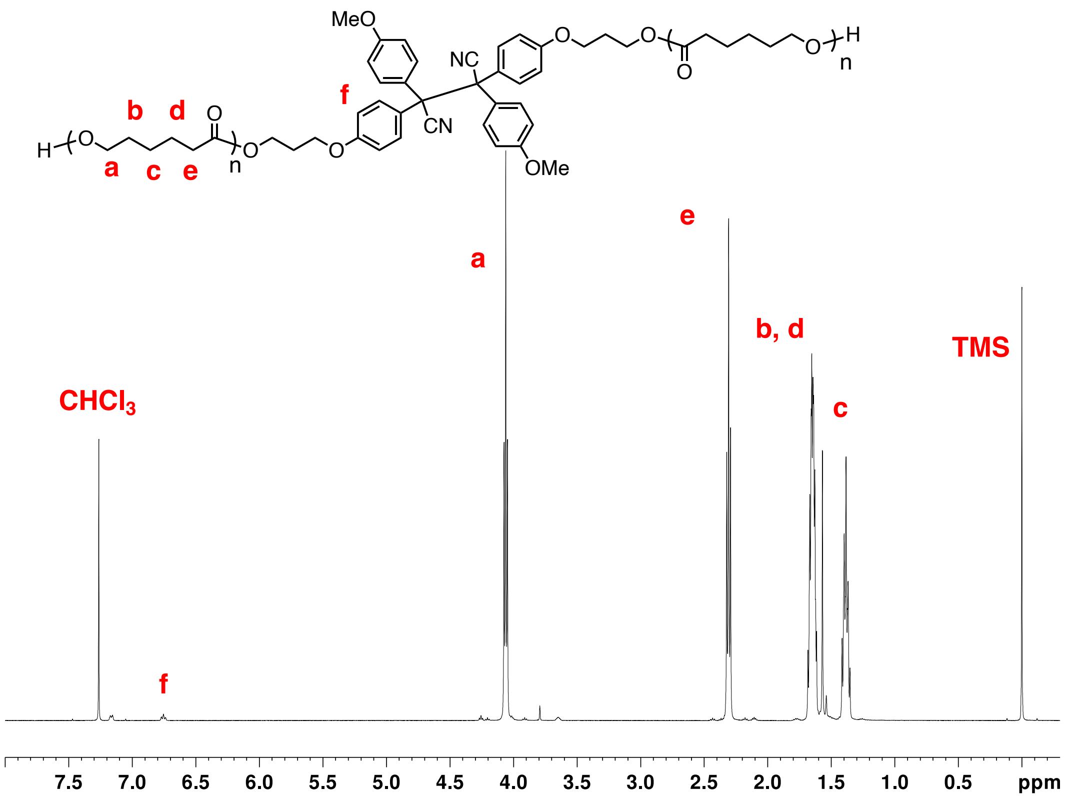

Supplementary Fig. 14.
^{1}H NMR spectrum of L-PCL ($M_{n,NMR}$ = 24400, M_w/M_n = 1.20). ($CDCl_3$, 500 MHz)

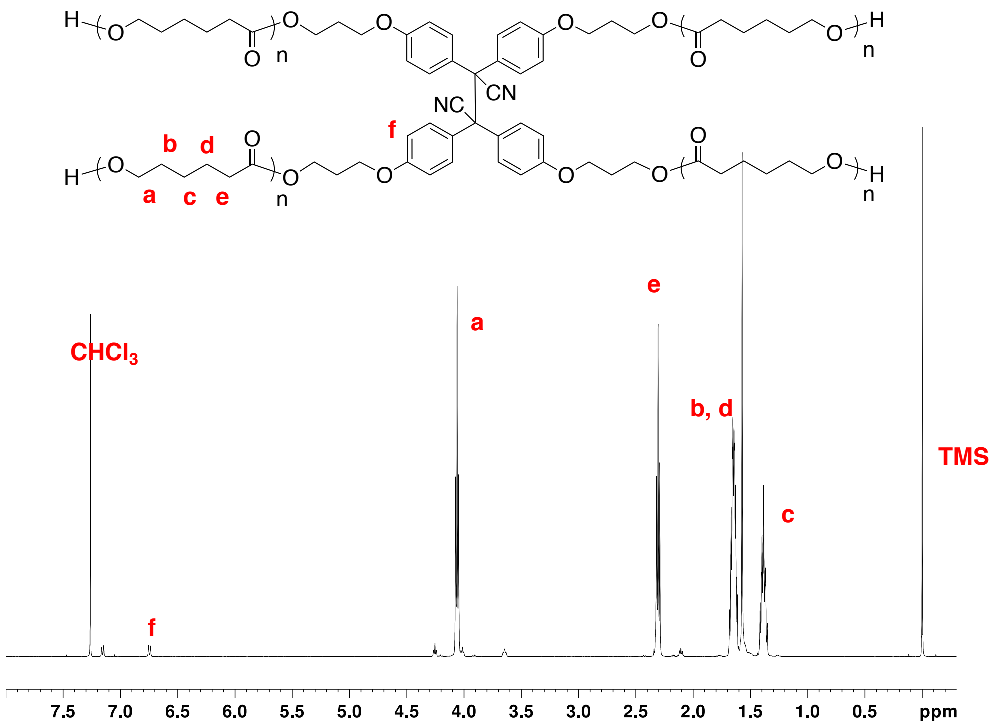

Supplementary Fig. 15.
^{1}H NMR spectrum of S-PCL ($M_{n,NMR}$ = 12600, M_w/M_n = 1.13). ($CDCl_3$, 500 MHz)

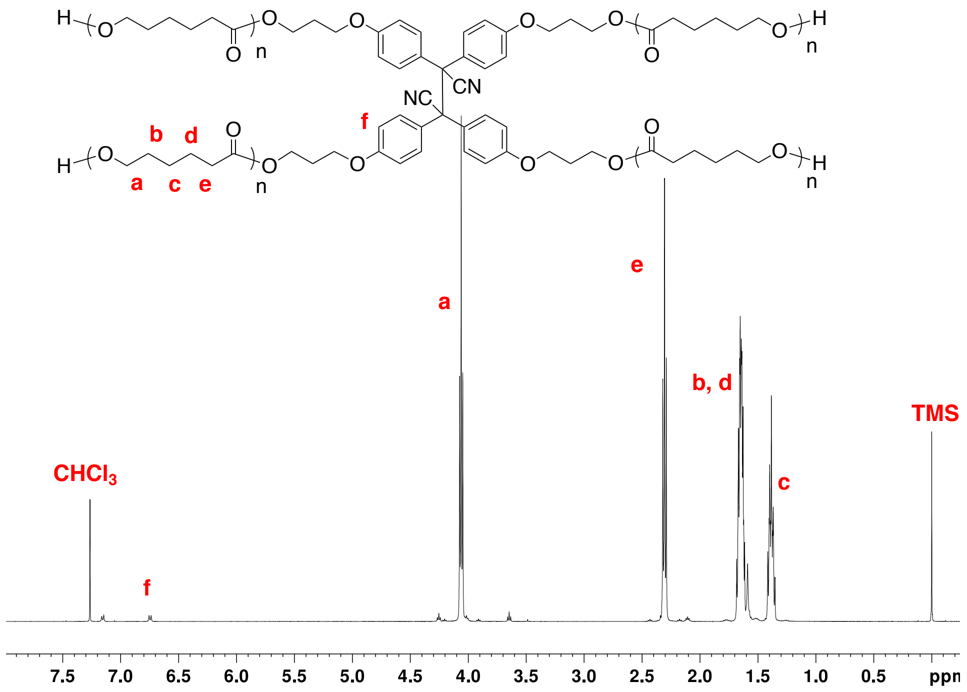

Supplementary Fig. 16.
^{1}H NMR spectrum of S-PCL ($M_{n,NMR}$ = 25200, M_w/M_n = 1.14). ($CDCl_3$, 500 MHz)

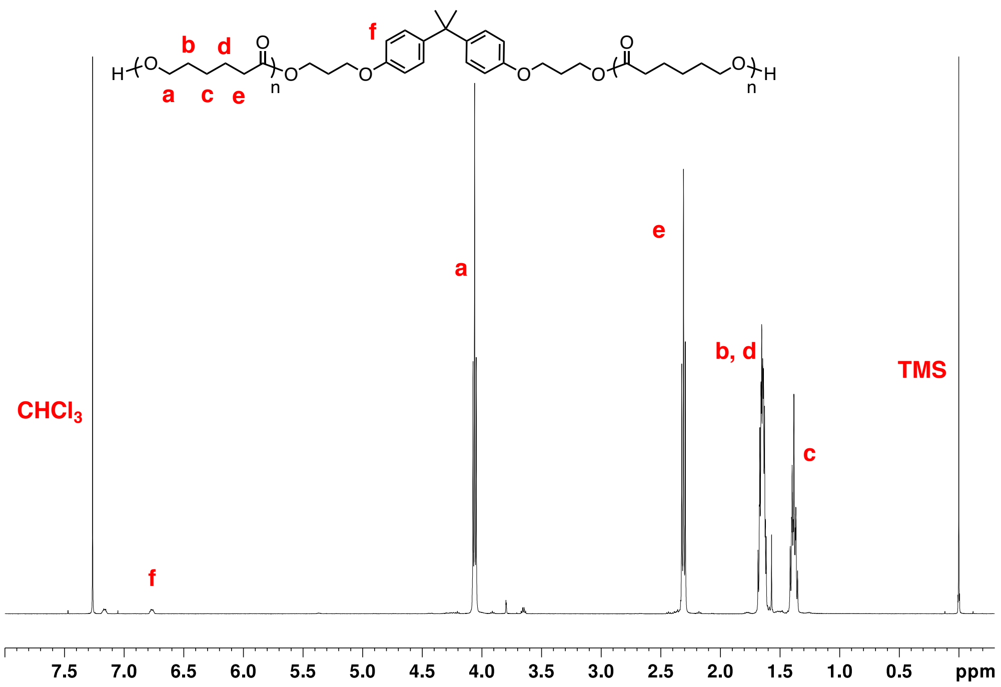

Supplementary Fig. 17.

^{1}H NMR spectrum of C_1-PCL ($M_{n,NMR}$ = 11800, M_w/M_n = 1.07). ($CDCl_3$, 500 MHz)

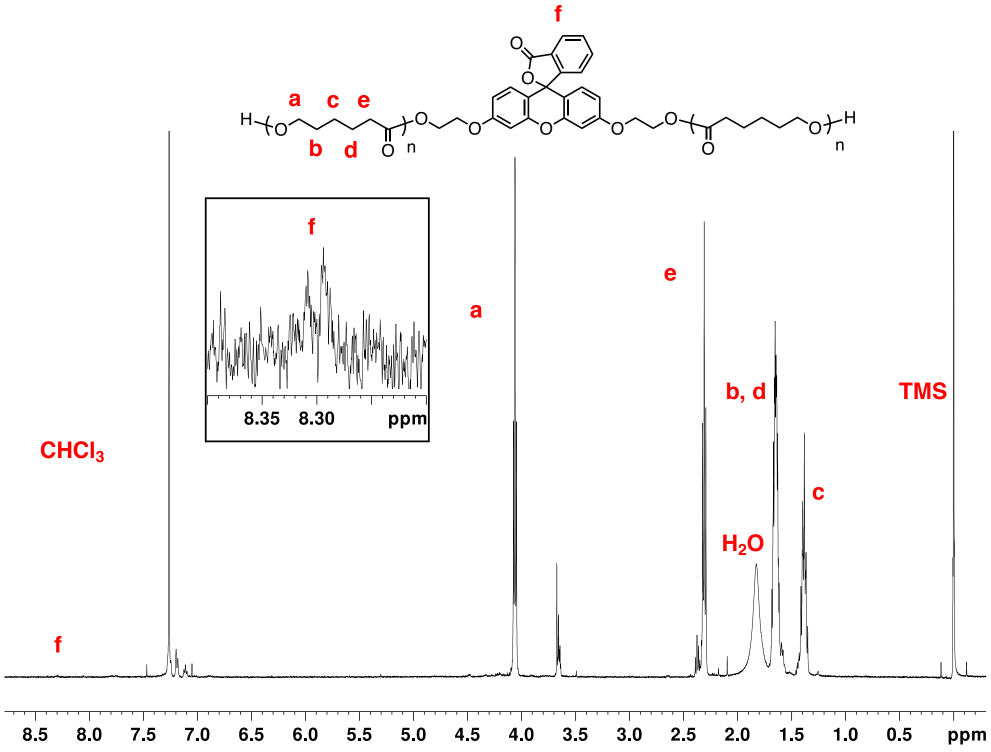

Supplementary Fig. 18.

^{1}H NMR spectrum of C_2-PCL ($M_{n,NMR}$ = 8600, M_w/M_n = 1.19). ($CDCl_3$, 500 MHz)

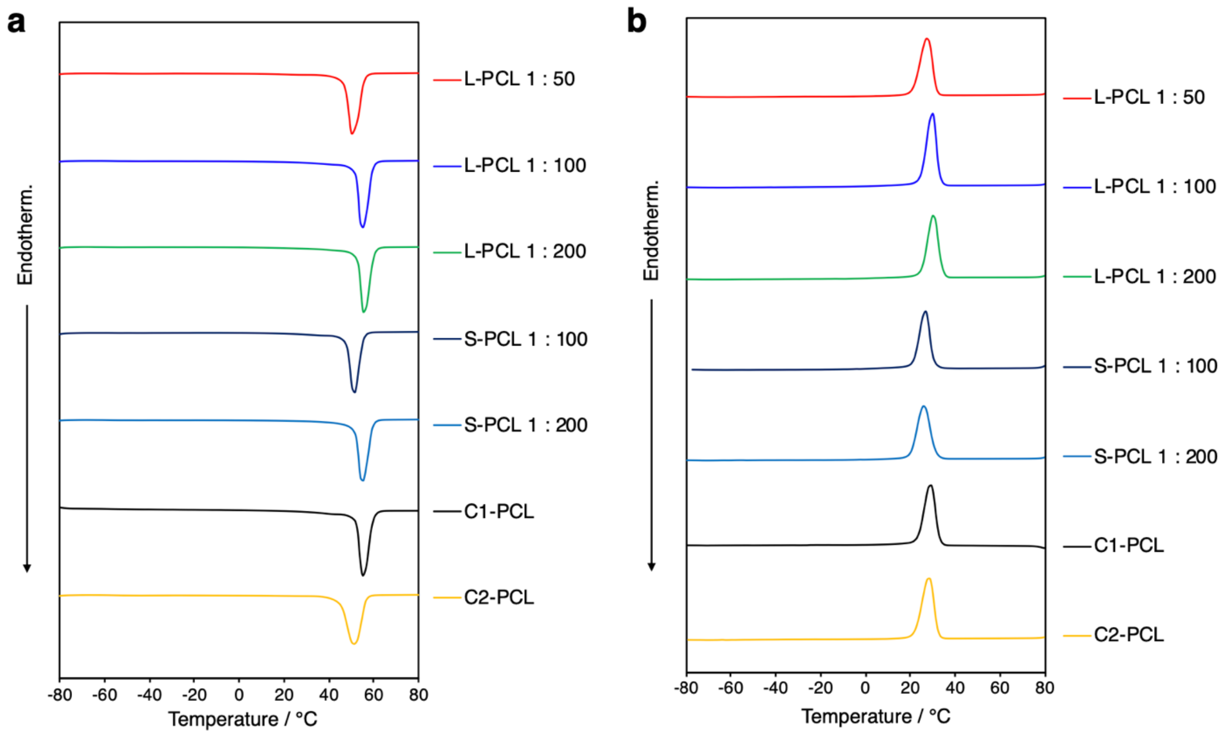

Supplementary Fig. 19.
DSC charts of crystalline polymer. DSC charts of **L-PCL**, **S-PCL**, **C_1-PCL** and **C_2-PCL** in (**a**) heating and (**b**) cooling process.

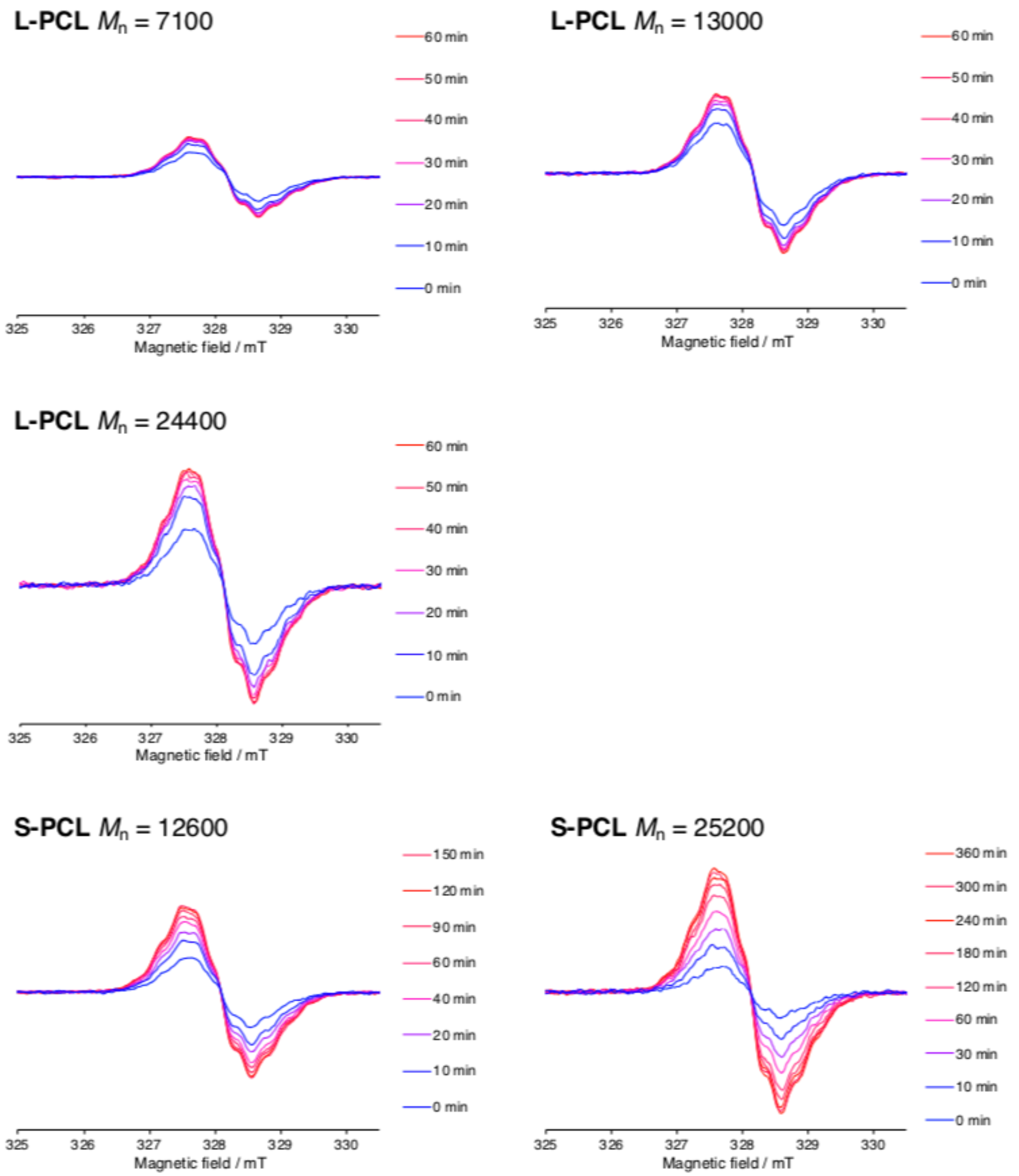

Supplementary Fig. 20.
Electron paramagnetic resonance spectra for crystalline polymer. EPR spectra of **L-PCL** ($M_{n,NMR}$ = 7100, 13000 and 24400) and **S-PCL** ($M_{n,NMR}$ = 12600 and 25200).

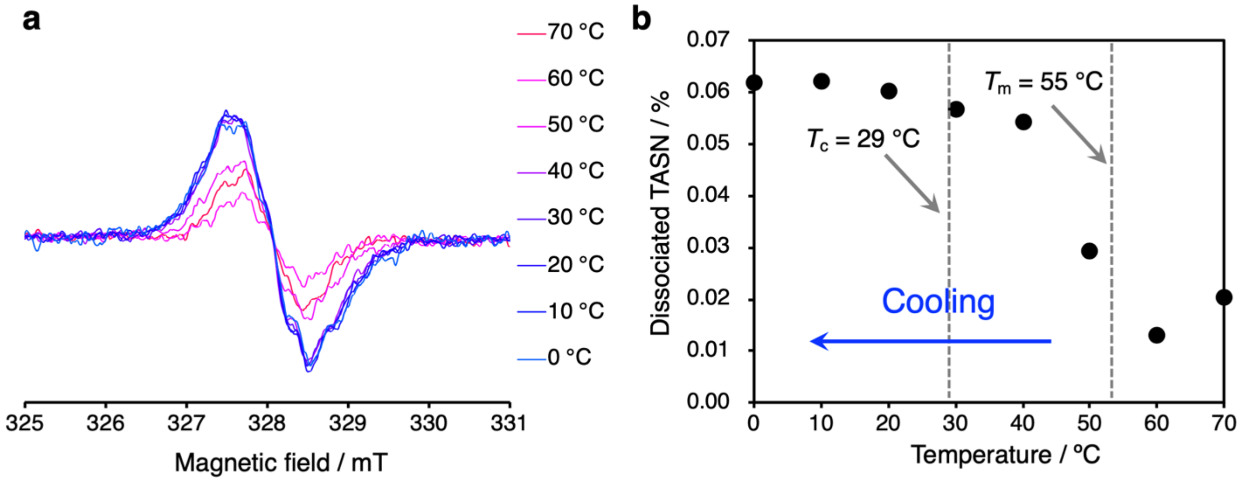

Supplementary Fig. 21.
EPR spectra of crystalline polymer in cooling process. (**a**) EPR spectra and (**b**) dissociated ratio of TASN moiety of **L-PCL** ($M_{n,NMR}$ = 13000) in cooling process from 70 °C to 0 °C.

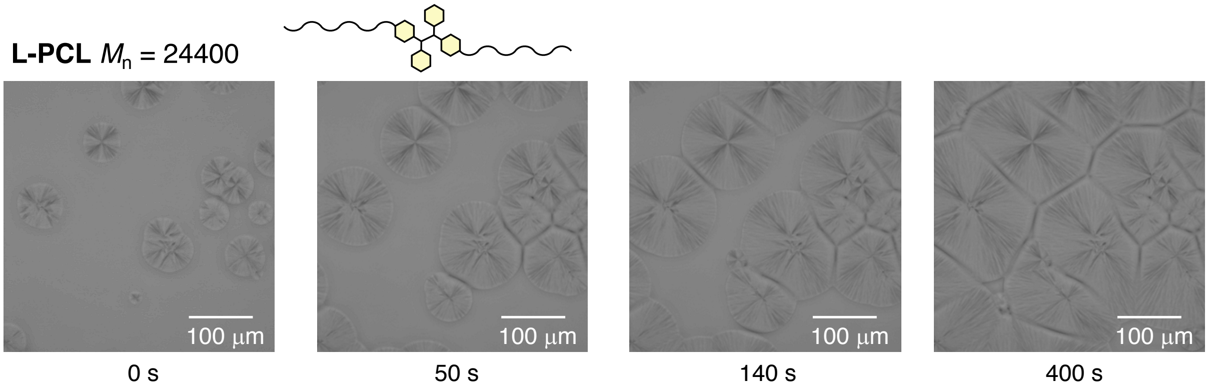

Supplementary Fig. 22.
Microscopic 2D image of L-PCL (M_n = 24400). Microscopic images of **L-PCL** (M_n = 24400) with 100 µm scale bar in isothermal crystallization.

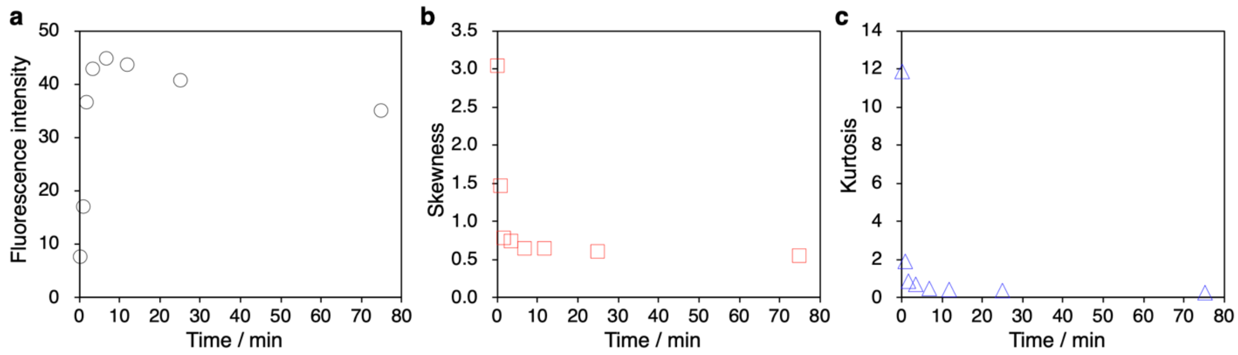

Supplementary Fig. 23.

Fluorescence data from 2D image of L-PCL (*M*$_n$ = 24400). (**a**) Fluorescence intensity, (**b**) skewness, and (**c**) Kurtosis of **L-PCL** (*M*$_n$ = 24400) by fluorescence microscope under 514 nm excitation with 100 μm scale bar in isothermal crystallization.

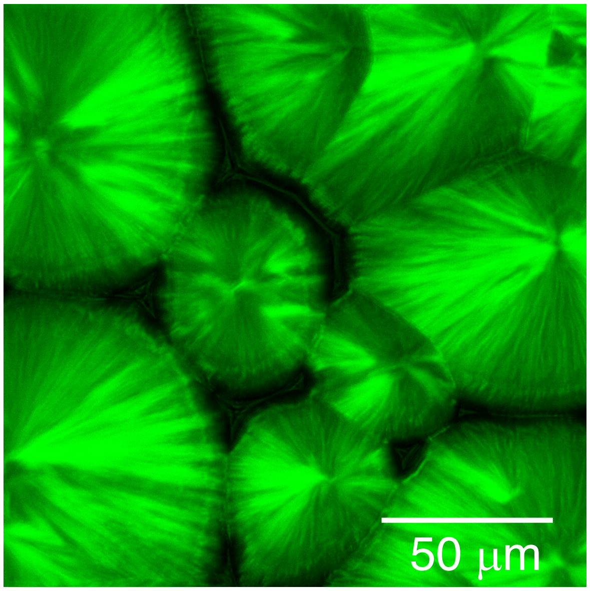

Supplementary Fig. 24.

CLSM 2D image in airy scan mode. Z scan mode of **L-PCL** (*M*$_n$ = 24400) under 514 nm excitation after isothermal crystallization in airy scan mode.

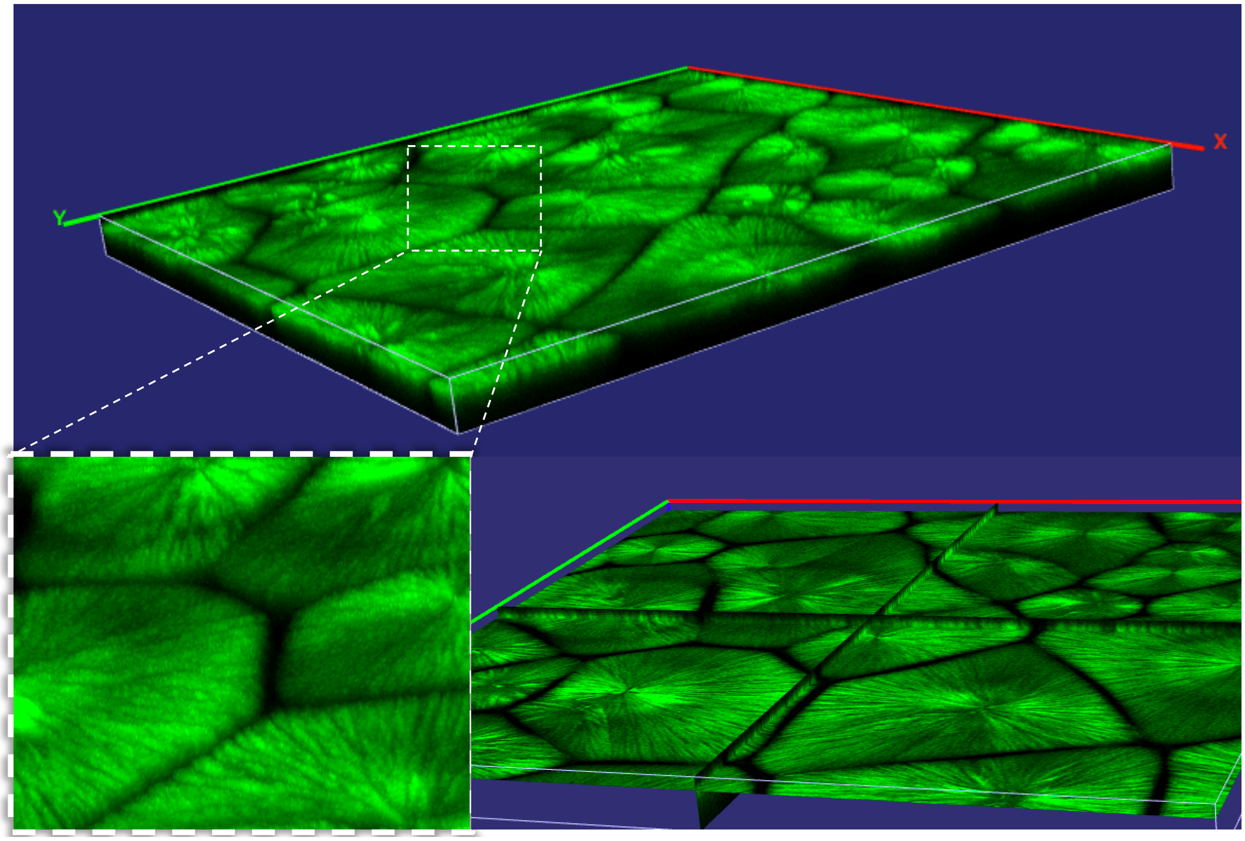

Supplementary Fig. 25.

CLSM 3D image under spin-coating condition (29 µm). Z scan mode of **L-PCL** (M_n = 24400) under 514 nm excitation after isothermal crystallization.

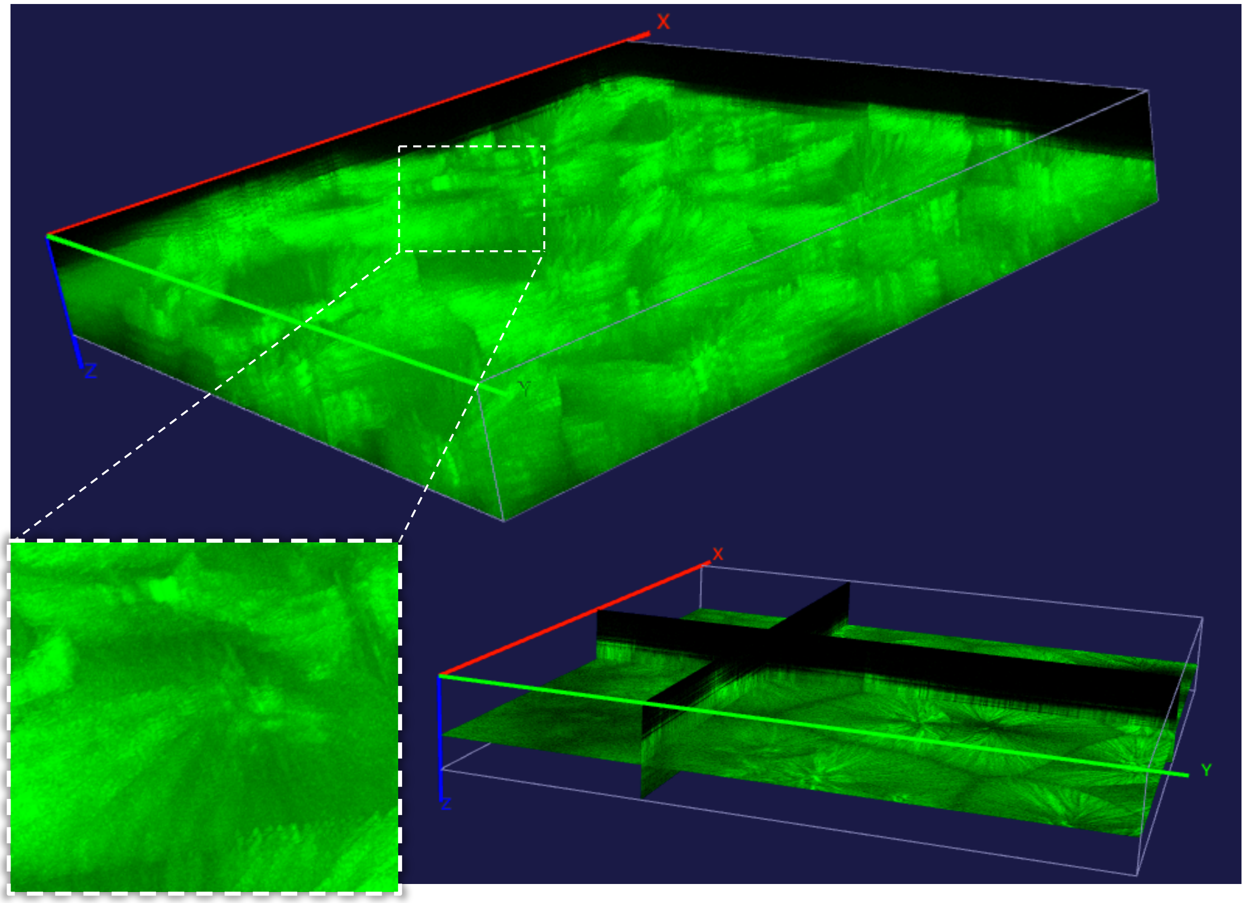

Supplementary Fig. 26.

CLSM 3D image under non spin-coating condition (90 μm). Z scan mode of **L-PCL** (M_n = 24400) under 514 nm excitation after isothermal crystallization.

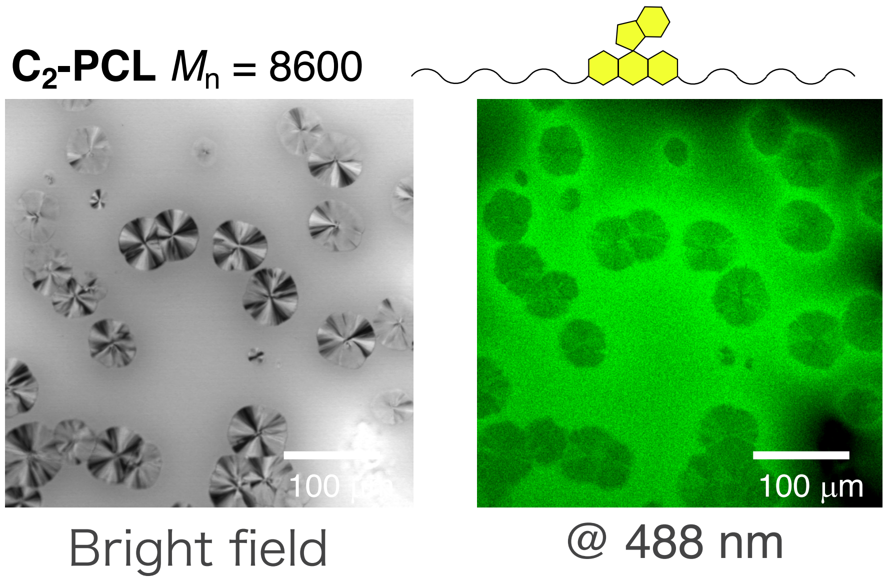

Supplementary Fig. 27.

CLSM 2D image of C_2-PCL. Microscopic images of **C_2-PCL** (M_n = 8600) under bright field condition (left) and fluorescence microscope under 488 nm excitation (right) in isothermal crystallization.

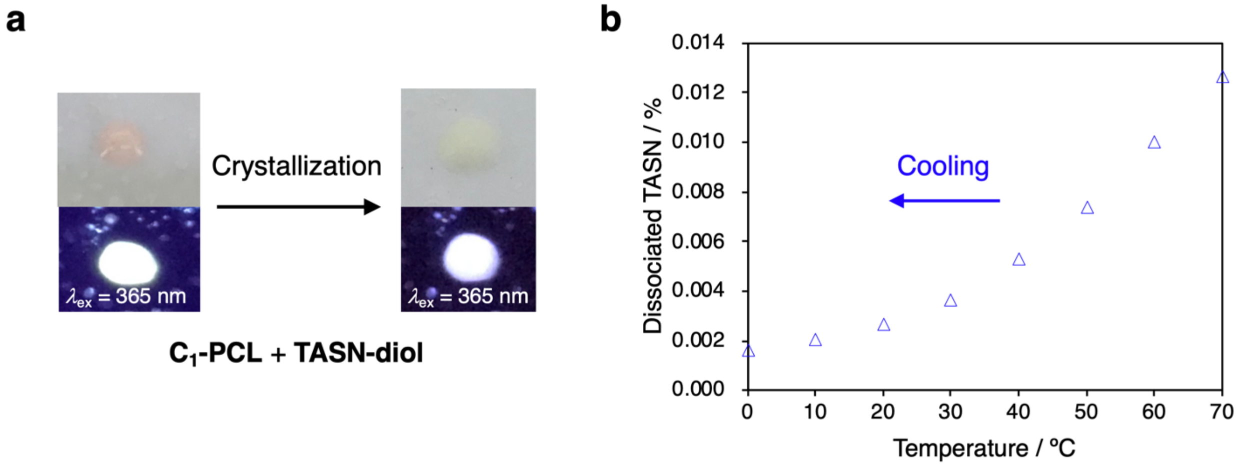

Supplementary Fig. 28.

Control experiments. (**a**) Photograph of **C_1-PCL** + **TASN-diol** (left) before and (right) after crystallization. (**b**) Dissociated TASN (%) in **C_1-PCL** + **TASN-diol** in cooling process.

Supplementary References

1 Sumi, T., Goseki, R. & Otsuka, H. Tetraarylsuccinonitriles as mechanochromophores to generate highly stable luminescent carbon-centered radicals. *Chem. Commun.* **53**, 11885-11888 (2017).

2 Kosuge, T. *et al.* Multicolor Mechanochromism of a Polymer/Silica Composite with Dual Distinct Mechanophores. *J. Am. Chem. Soc.* **141**, 1898-1902 (2019).
