# Supplementary material for: Crystallization-induced mechanofluorescence for visualization of polymer crystallization
Source: Nat Commun. 2021 Jan 5;12:126. doi: 10.1038/s41467-020-20366-y (PMC7785725; doi:10.1038/s41467-020-20366-y)
Supplement: Supplementary file 2 — Description of Additional Supplementary Files [file 41467_2020_20366_MOESM2_ESM.pdf]

Description of Additional Supplementary Files

File Name: Supplementary Movie 1

Description**: Video of microscopic 2D image of L-PCL (Mn = 24400).** Video of microscopic images of L-PCL (Mn = 24400) during isothermal crystallization captured (left) in bright field and (right) by confocal laser scanning microscopy (CLSM; λex = 514 nm).
